# Chirality in Transition Metal Dichalcogenide Nanostructures

**DOI:** 10.1002/chem.202404765

**Published:** 2025-05-26

**Authors:** Lorenzo Branzi, Joseph Martyn, Lucy Fitzsimmons, Yurii K. Gun'ko

**Affiliations:** ^1^ School of Chemistry CRANN and AMBER Research Centers Trinity College Dublin College Green Dublin 2 Ireland

**Keywords:** chirality, molybdenum disulfide, nanostructures, nanotechnology, transition metal dichalcogenides

## Abstract

The fascinating properties introduced by the breaking of mirror symmetry have recently motivated a rising interest in chirality in nanomaterials. In particular, transition metal (TM) dichalcogenides (TMDs) are a wide group of technologically relevant 2D layered materials where recent efforts in the introduction of chirality have shown promising results, attracting great attention for future studies and potential applications. This review article is focused on the development of chirality in TM dichalcogenide nanostructures, dealing with the synthetic strategies that have been adopted to produce chiral TMDs both via solution‐phase and vapor‐phase syntheses along with the characterization of their chiroptical properties. A broad range of examples, including a variety of nanostructures such as 0D quantum dots (QDs), 1D nanotubes, 2D flakes, and more complex 3D nanostructures as well as different origins of chirality are considered. Critical analysis of potential pitfalls in the assessment of the materials’ chirality are discussed. A broad range of exciting properties and applications associated with the materials’ chirality, including: nanomedicine, enantioselective catalysis, spin‐dependent electrocatalysis, spintronics, and nonlinear optics, are also presented in the review.

## Graphical abstract

The emergence of chirality in inorganic nanostructures has a critical impact on the properties and potential applications of these novel nanomaterials. This work presents a critical collection of examples of the observation of chirality in TMDs, covering different synthetic procedures and origins of chirality as well as a broad range of dimensionality from 0D dots to complex 3D nanostructures.

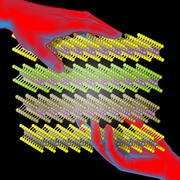

## 1 Introduction

The phenomenon of chirality is a ubiquitous feature in materials chemistry, from the stereochemistry of simple molecules to complex assemblies in the microscale and spreading across a broad range of molecular clusters and nanostructures.^[^ ^1^, ^2^, ^3^ ^]^ The investigation of chirality in nanomaterials is heavily motivated by an interest in discovering novel materials with unique properties that originate from the breaking of mirror symmetry or from the presence of chiral interfaces, which grant enantioselective interactions with other chiral systems.^[^ ^4^, ^5^, ^6^, ^7^, ^8^, ^9^ ^]^ Furthermore, fundamental questions such as the investigation of the origin of homochirality in biological systems and the constant interest in novel synthetic approaches that enable precise control of crystal growth of nano‐ and microstructures have garnered interest in this field in recent years.^[^ ^10^, ^11^, ^12^ ^]^

Transition metal dichalcogenides (TMDs) are a class of 2D layered material characterized by the general formula MX_2_ where M is a transition metal (TM) and X is a chalcogen atom. They display a number of interesting properties, which set them apart from other layered materials. They exhibit multiple crystallographic phases, dependent on the stacking of their monolayers within their crystal structure and differing TM coordination environments, resulting in phases with a broad range of electronic properties ranging from semiconducting to semimetallic and metallic behavior.^[^ ^13^ ^]^ Bonding between TMD layers is dictated by weak van der Waals interactions, which allow for their facile separation via a variety of top‐down techniques. In addition to this, TMD nanomaterials can be readily grown by bottom‐up synthetic strategies including hot injection, solvothermal synthesis, and chemical vapor deposition (CVD).^[^ ^14^ ^]^ The variety in choice of potential synthetic approaches and crystallographic phases has led to the production of a vast library of TMD nanostructures, such as nanosheets, nanorods, nanoflowers, and QDs, with varying electronic, optical, and mechanical properties. Chiral TMD‐based nanomaterials hold great potential due to their unique structural and electronic properties. Their chirality introduces asymmetric interactions, enabling advanced functionalities in areas such as optoelectronics, spintronics, and enantioselective catalysis. These materials can also manipulate polarized light and electron spin in ways that conventional materials cannot, offering pathways to develop next‐generation devices with high efficiency and precision. Moreover, chiral TMDs could be critical for applications in sensing, biomedicine, and energy conversion, where selective interactions at the nanoscale are essential. Therefore, understanding and harnessing chirality in TMD nanomaterials is very important and could result in new cutting‐edge technologies and materials.

This review article is focused on advancements in the synthesis of chiral TMD nanostructures, their properties, and related applications. A short introduction is dedicated to the basic concept of chirality in nanomaterials and its different origins, followed by an overview of TMD structure and properties, and the synthetic methods used for the production of TMD‐based nanostructures. The main text is focused on different examples of the emergence of chirality in TMDs nanostructures produced via both solution and vapor‐phase syntheses. Particular attention is dedicated to synthetic aspects and the characterization of the chiroptical properties of chiral TMD nanostructures and the origin of the symmetry breaking. Applications and properties of the different chiral TMD nanostructures are addressed in depth case‐by‐case in the text. In particular, we divide the main section between nanostructures produced by solution‐phase and vapor‐phase syntheses. The former is focused on TMD nanostructures produced by crystal growth starting from a homogeneous solution or processed in a way to give a colloidal dispersion. The latter is focused on the production of TMD flakes or 1D nanotubes via vapor‐to‐solid growth approaches like CVD and chemical vapor transfer. The induction of chiral features in both systems shows promising properties and opens novel possibilities for potential applications. This manuscript is the first attempt to rationalize the emergence of chirality in TMD nanostructures, offering a unique point of view on this fast‐growing field with a broad range of examples spanning from 0D to 3D nanostructures along with a broad range of examples of different origins of chirality.

### 1.1 Chirality in Inorganic Nanostructures

As defined by IUPAC, chirality is “the geometric property of a rigid object (or spatial arrangement of points or atoms) of being nonsuperposable on its mirror image.”^[^ ^15^ ^]^ These pairs of mirror images, known as enantiomers, possess identical physical and chemical characteristics, but are distinguishable through their interactions with other chiral objects and with polarized light. While most often used to describe organic small molecules and biomolecules, chirality is a geometric property not necessarily related to the size of the object being described.^[^ ^1^ ^]^ Accordingly, in recent decades chirality and chiroptical activity have been demonstrated in a wide range of nanomaterials, such as QDs, (e.g., CdS nanocrystals),^[^ ^16^, ^17^ ^]^ transition metal oxides,^[^ ^18^ ^]^ and plasmonic metal nanoparticles (NPs).^[^ ^6^ ^]^ Chirality in nanostructures is mostly probed via spectropolarimetry, in particular, Circular Dichroism (CD) spectroscopy is the main technique that is used for the evaluation of the material's chirality. This technique relies on the differential absorption of circularly polarized light with different handedness, revealing critical insight on the symmetry breaking. The use of CD spectroscopy for this purpose has been discussed in detail by several authors before.^[^ ^19^ ^]^ CD spectra of nanomaterials are typically reported as ellipticity (expressed as degrees or millidegrees) of the light after passing through the sample. The relationship between the difference in absorption of left‐and right‐polarized light ΔA and the ellipticity Θ is given by:

(1) $$\Delta A=A_{L}-A_{R}=\theta /32982$$

where $A_{L}$ and $A_{R}$ are the absorbances of left‐and right‐circularly polarized light, respectively. The rotational strength R associated with a single electronic transition from an initial state (i) to a final state (f) represents the integrated intensity of the CD band and depends on the imaginary part of the dot product between the electronic ($\vec{\mu }$) and magnetic ($\vec{m}$) transition dipole moments as:

(2) $$R_{\mathrm{if}}=Im\left\{{\vec{\mu }}_{\mathrm{if}}\cdot {\vec{m}}_{\mathrm{if}}\right\}$$

For a generic chiral colloidal nanosystem, produced using a chiral ligand, the CD spectra can be associated with one of these three scenarios: 1) the CD signal is largely dominated by the chiral ligand contribution, potentially showing different intensities and/or line shapes compared to the free ligand due to hybridization with material surface states, and no optical activity is observed in the region where the material itself absorbs light. This is typically the case of hybrid systems where the chirality is retained in the chiral ligand with little or no effect on the inorganic phase. 2) CD bands are observed corresponding to the electronic transitions of both the ligand and the inorganic phase. This situation is typically associated with a chirality transfer producing a breaking of the mirror symmetry in the electronic transition of the inorganic phase. Finally, 3) the intensity of the CD bands observed in the case of nanostructures exhibiting chiral morphology is usually independent of the ligand‐related transitions, which in some cases are not observed. Spectroscopic data can, in some cases, be used for the evaluation of the g‐factor, a dimensionless factor that allows a simple comparison of sign and magnitude of the optical activity.^[^ ^20^ ^]^ The g‐factor is calculated simply as the difference between Δ*A* and the absorption of nonpolarized light (A) a given wavelength, given by Equation 3 and covers a range of values from +2 to ‐2. Usual values observed for nanomaterials are strongly dependent on the origin of the chirality, spanning from 10^−4^ for ligand‐induced chirality to 10^−1^ for nanoparticles with complex chiral morphologies.^[^ ^2^ ^]^

(3) $$\Delta A/A=(A_{L}-A_{R})/A$$

However, chiroptical properties may not always be useful to disclose chirality. Several conditions could lead to the cancellation of the CD signal of the ensemble, such as the presence of a complex mixture of diastereoisomers or a perfect racemate of two enantiomers.^[^ ^21^, ^22^ ^]^ The analysis of the chiroptical activity of single particles can distinguish chirality in overall achiral systems, for example, the use of circularly polarized luminescence microscopy for the determination of chirality for individual TbPO_4_ nanocrystals.^[^ ^23^ ^]^ The lack of electronic transitions is another limitation of the optical approaches, especially for insulating materials and wide band gap semiconductors. Isothermal titration calorimetry (ITC) has proven to be a useful tool for the assessment of chirality in different systems such as zeolites,^[^ ^24^ ^]^ nanoporous carbon,^[^ ^25^ ^]^ and silica nanotubes.^[^ ^26^ ^]^ Moreover, ITC data are more informative on the interface chirality, providing a more detailed view of enantioselective interactions on the nanomaterial surface, which is particularly relevant for application in sensing, separation, and catalysis.

**Figure 1:**
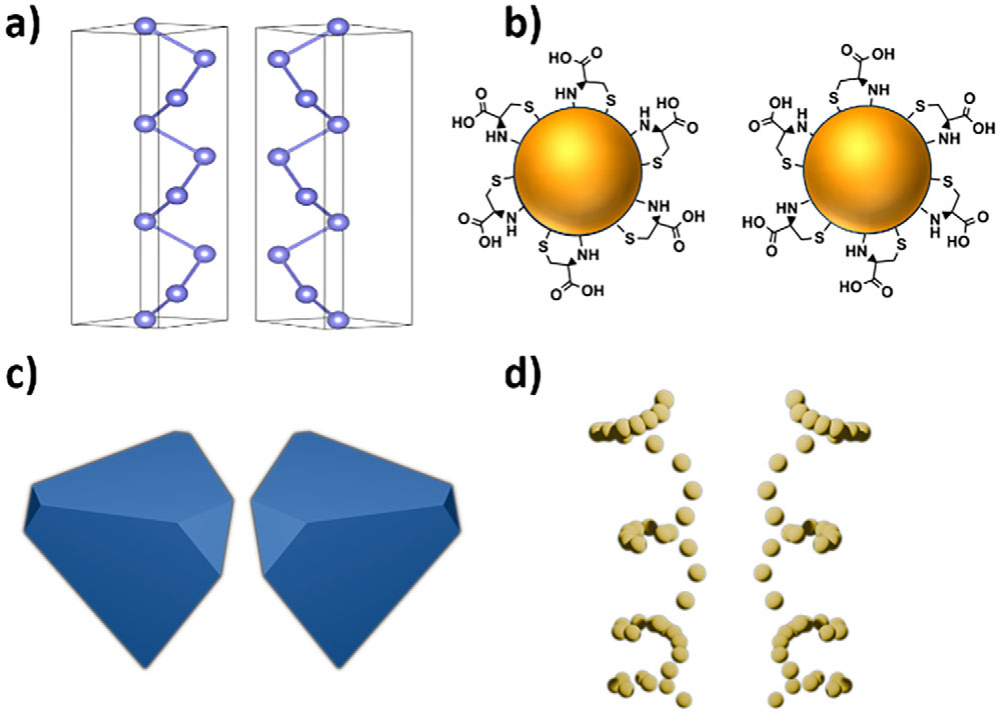
Types of chirality in inorganic nanostructures. a) Enantiomorphs of tellurium exhibiting structural chirality, b) ligand‐induced chirality using cysteine as chiral ligand, c) asymmetric tetrahedra showcasing morphological chirality, and d) chiral helical assemblies of nanoparticles.

Chirality in nanomaterials and nanostructures is associated with a large variety of origins, which can be broadly broken down into intrinsic chirality (Figure 1a), ligand‐induced chirality (Figure 1b), morphological chirality (Figure 1c), and chiral assembly (Figure 1d). Intrinsic chirality results from an intrinsically chiral crystal structure. For example, Ben‐Moshe et al.^[^ ^27^ ^]^ used the chiral ligand penicillamine to selectively form one enantiomer of inherently chiral *α*‐HgS nanocrystals (NCs) from achiral *β*‐HgS, which exhibited remarkably high optical activity. Despite the high optical activity of intrinsically chiral nanomaterials, this method is inherently limited in scope, as only 65 space groups out of 230 overall are chiral, these are called the Sohncke groups.^[^ ^28^ ^]^ Among these, 22 are enantiomorphic and divided into 11 pairs, the remaining 43 are low‐symmetry space groups. Relevant materials crystallizing in these space groups include quartz, *β*‐Ag_2_Se, Se, and Te (Figure 1a).^[^ ^29^, ^30^, ^31^, ^32^ ^]^

**Figure 2:**
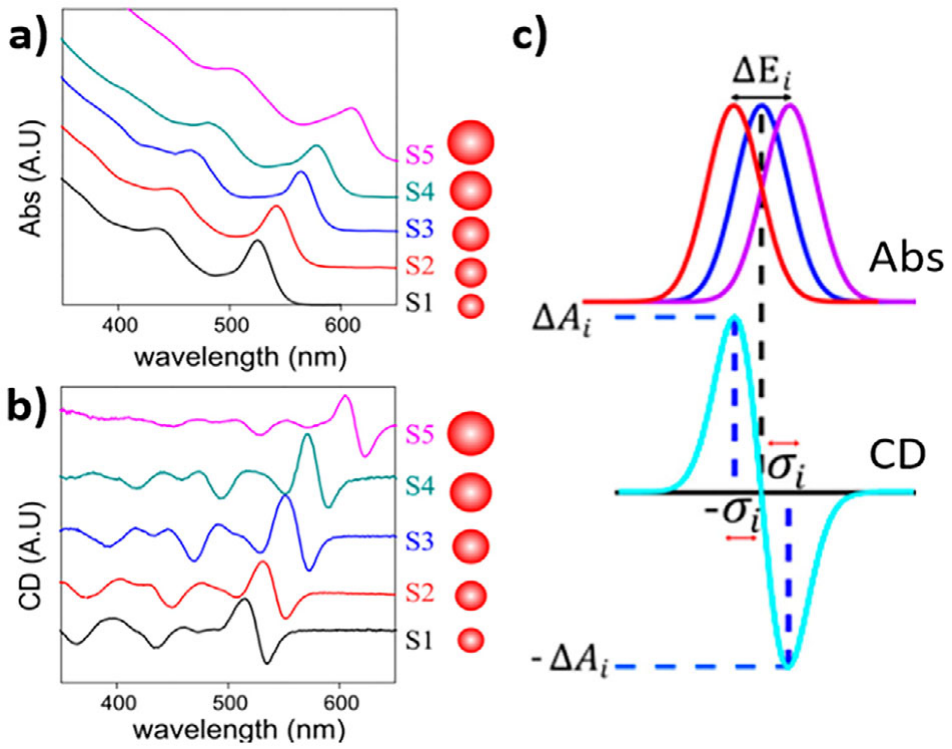
Absorption a) and CD b) spectra of cysteine‐functionalized CdSe QDs of sizes 2.6–5.3 nm, showcasing characteristic derivative signal shape at exciton band. c) Schematic depicting calculation of energy splitting (ΔE_i_) from absorption and CD data. Adapted with permission from Ben‐Moshe et al.^[^ ^33^ ^]^

Chirality can originate at the particle‐ligand interface from the interaction between a chiral ligand and an achiral NP. In these hybrid systems, chiroptical activity can be introduced in the NP characteristic electronic transitions (e.g., plasmonic or excitonic) through electronic coupling and/or structural distortion.^[^ ^33^ ^]^ Chiroptical activity resulting from functionalization with chiral ligands has been demonstrated in a wide range of materials, particularly in Au NPs and QDs. Semiconductor NCs functionalized with chiral ligands are among the most‐studied systems of chiral nanomaterials, with chiroptical activity demonstrated in a wide range of materials of monoelemental (e.g., Si),^[^ ^34^ ^]^ binary (e.g., CdS, CdSe),^[^ ^16^, ^35^ ^]^ and ternary composition (e.g., silver indium sulfide).^[^ ^36^ ^]^ The chiral ligand can induce chirality in the achiral nanoparticles via both electronic coupling and structural distortions, and a range of models have been proposed to explain the transfer of chirality in ligand‐induced systems, particularly in QDs. A commonly used description of chiroptical activity in biomolecule‐functionalized plasmonic NPs was developed by Govorov et al.^[^ ^19^ ^]^ The induction of chiroptical activity in plasmonic metal NPs was ascribed to chiral currents generated by the oscillating dipole of the chiral molecule. However, the magnitude of CD induced by this mechanism is strongly dependent on the material's dielectric constant, and thus this model is typically not applicable to semiconductor NPs. Ben‐Moshe et al.^[^ ^33^ ^]^ used UV‐Visible (UV‐Vis) and CD spectroscopy to examine ligand‐QD interactions in CdS and CdSe QDs prepared via hot injection followed by phase transfer into aqueous cysteine solution. The authors found that excitonic transitions visible in absorption spectra were accompanied by characteristic derivative CD signals (Figure 2a,b), indicating that the induced CD spectra were due to splitting of the hole levels into two sub‐bands, each with preferential excitation by left‐ and right‐handed polarized light (Figure 2c).

**Figure 3:**
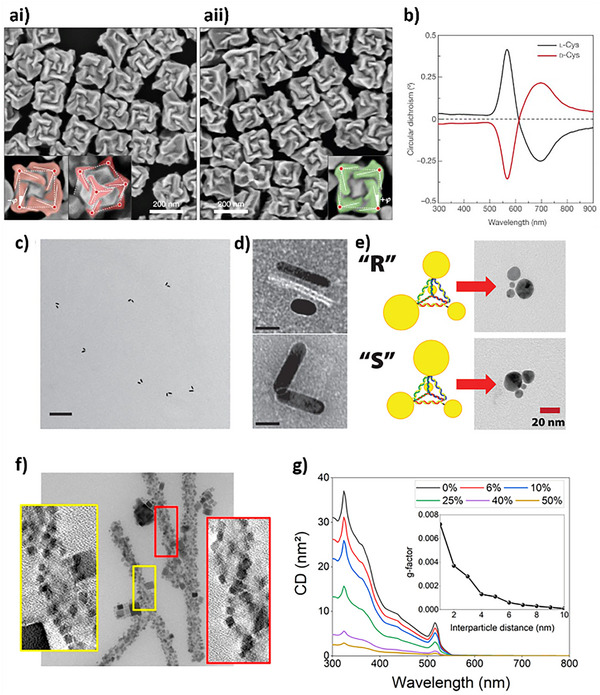
a) Scanning electron microscope (SEM) images of b) L‐ and c) D‐cysteine‐functionalized Au NPs. Insets indicate tilting of vertices with respect to cubic outline (dotted line) and vertices (red dots) as viewed along [100] (ai, left) and [111] (ai, right) directions. b) CD spectra of L‐ and D‐cysteine‐functionalized plasmonic Au NPs. Examples of chiral nanoassemblies. c) TEM images of chiral Au NR dimers on DNA origami (scale bar: 200 nm). d) Enlarged TEM images of chiral Au NR dimers from different angles (scale bars: 20 nm). e) Schematics and TEM images of chiral nanopyramids formed from Au NPs linked by DNA. f) TEM images of nanohelical assemblies of CsPbBr_3_ NCs on helical silica nanoribbons and g) simulated effect on CD spectra of CsPbBr_3_ NCs of random removal of NCs. Inset: evolution of g‐factor of increased interparticle distance through removal of 30% of NCs. Au NPs. Figure a,b) are adapted with permission from Lee et al.,^[^ ^12^ ^]^ c,d) adapted with permission from Zhou et al.,^[^ ^41^ ^]^ e) adapted with permission from Mastroianni et al.,^[^ ^42^ ^]^ and f,g) adapted with permission from Liu et al.^[^ ^43^ ^]^

Chirality can also be introduced into nanomaterials by the formation of nano‐ and microcrystals with chiral morphology. Intense chiroptical activity has been observed through the formation of highly anisotropic structures such as helices,^[^ ^37^, ^38^ ^]^ helicoids,^[^ ^12^ ^]^ twisted nanorods (NR),^[^ ^39^ ^]^ and truncated tetrahedra.^[^ ^40^ ^]^ Growth of these chiral morphologies can be achieved through a variety of methods, such as illumination by circularly polarized light,^[^ ^40^ ^]^ use of chiral ligands,^[^ ^12^ ^]^ and templating on chiral precursors (e.g., chiral silica nanoribbons).^[^ ^37^ ^]^ Lee et al.^[^ ^12^ ^]^ used cysteine and cysteine‐derived peptides to aid the growth of high Miller index surfaces from achiral seed crystals with exposed low‐index planes. This approach afforded highly asymmetric crystals (Figure 3a) exhibiting intense chiroptical activity (Figure 3b), with asymmetry factors (*g*‐factors) as high as 0.2.

Chiral assemblies of achiral NPs, such as helices,^[^ ^40^, ^44^, ^45^, ^46^ ^]^ nanorod dimers (Figure 3c,d),^[^ ^41^, ^47^ ^]^ and asymmetric pyramids (Figure 3e),^[^ ^42^, ^48^ ^]^ can also exhibit chiroptical activity. Nanohelical assemblies are among the most well‐investigated examples, most commonly formed through the self‐assembly of NPs with biomacromolecules such as DNA, DNA origami, and peptides.^[^ ^42^, ^44^, ^49^ ^]^ Inorganic templates are also possible through the self‐assembly of individual NPs with chiral surface ligands.^[^ ^45^ ^]^ Templating of materials through self‐assembly of chiral amphiphiles followed by removal of organics permits the bottom‐up synthesis of chiral all‐inorganic materials, most commonly twisted silica nanoribbons and mesoporous nanorods.^[^ ^50^, ^51^, ^52^ ^]^ Subsequent growth of nanoparticles on these templates has been used to produce chiral inorganic assemblies of a wide variety of materials.^[^ ^43^, ^46^, ^53^ ^]^ Oda and coworkers^[^ ^43^ ^]^ provided an explanation for the origin of chiroptical activity in semiconductor nanoassemblies by examining the effect of interparticle distance on the CD spectra of CsPbBr_3_ perovskite NCs helically arranged on silica nanohelices (Figure 3f). The origin of the CD signal was ascribed to highly distance‐dependent dipolar interactions between perovskite NCs, with g‐factors decreasing over 90% when the interparticle distance was increased from 1 nm to 4 nm (Figure 3g). Experimentally, the correlation distance was tuned by varying drying time, and grazing incidence X‐ray scattering, and transmission electron microscopy (TEM) data showed excellent agreement with theory.

### 1.2 TMD Nanostructures

TMDs are materials of the form MX_2_ where M is a TM (e.g., Mo, W) and X is any chalcogenide (e.g., S, Se, Te). They are a key example of layered materials, made up of monolayer sheets where an atomic layer of the TM is sandwiched between two atomic layers of chalcogenide atoms. Bonding within individual monolayers is strong, but interlayer interactions are dictated by weak van der Waals forces, which allow for ease of separation of layers via a wide variety of mechanical and chemical procedures. One of their advantages as a 2D material is their structurally dependent electronic properties, with their polymorphs displaying both metallic, semimetallic, and semiconducting behavior.^[^ ^54^ ^]^

TMDs in both their bulk and nanocrystalline forms have been the subject of research since the mid‐20^th^ century.^[^ ^55^, ^56^ ^]^ However, the interest in layered materials such as TMDs surged in the wake of the graphene revolution of the early 2000s. This began in 2004 when Novoselov and Geim reported the so‐called Scotch Tape method, using mechanical cleavage for the facile production of monolayer graphene.^[^ ^57^ ^]^ Soon the field turned to more scalable methods of 2D nanomaterial production, particularly liquid‐phase exfoliation (LPE).^[^ ^58^, ^59^ ^]^ TMDs in particular were of significant interest due to their promising electronic properties; for example, they exhibit nonzero bandgaps in contrast to graphene, allowing for predicted potential use in post‐silicon field effect transistors.^[^ ^60^ ^]^ They also display a layer‐dependent band gap tunability: with a decreasing number of monolayers an indirect to direct transition is observed.^[^ ^61^, ^62^ ^]^

#### 1.2.1 Polymorphism in TMDs

TMDs occur in three main polymorphs, namely the 1T (trigonal), 2H (hexagonal), and 3R (rhombohedral) phases. This nomenclature arises from the number of stoichiometric TMD monolayers within the unit cell and the crystal family of the material.^[^ ^63^ ^]^ These polymorphs arise due to the difference in the TM coordination environments and the stacking of TMD monolayers.

**Figure 4:**
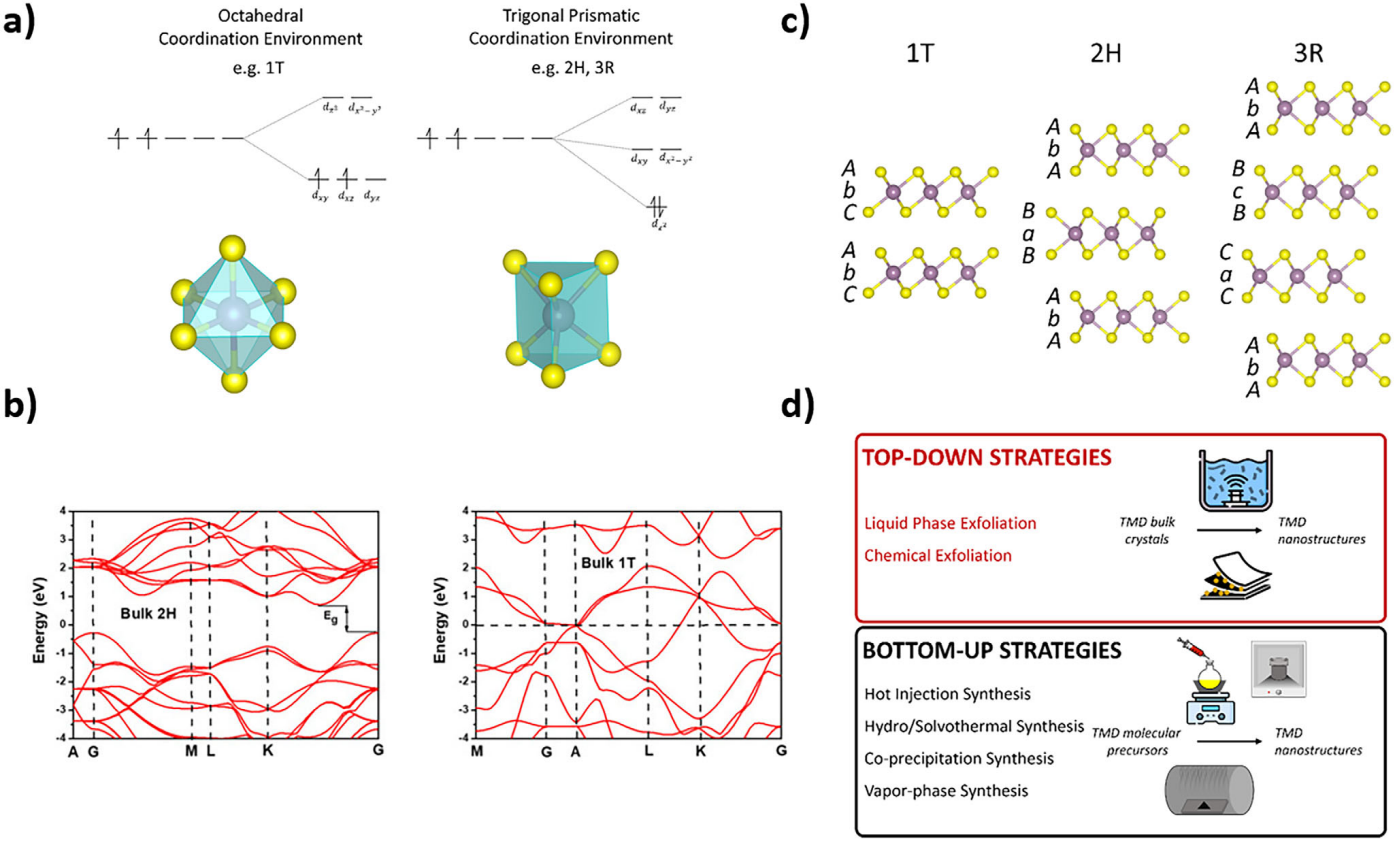
a) Schematic of TM coordination environments and resulting d‐orbital energy splitting diagrams b) schematic of layer stacking in TMD crystals of 1T, 2H, and 3R phases (COD 1 010 993, ICSD 254 956, and ICSD 38 401, respectively), c) band structure of 1T and 2H phases of TMD materials d) schematic of top‐down and bottom‐up approaches used for producing TMD nanostructures. Figure c adapted with permission from Zhao et al.^[^ ^64^ ^]^

The most common phase of TMDs, the 2H phase, belongs to the hexagonal (H) crystal system and exhibits *AbA BaB* stacking. Each TM atom is in a trigonal prismatic coordination environment. The 2H phase is semiconducting, with complete filling of its lowest energy orbital – dz^2 ‐^ due to the trigonal prismatic coordination of the TM (Figure 4a). It is an indirect bandgap semiconductor in the bulk form but undergoes a transition to a direct bandgap as a monolayer.^[^ ^61^ ^]^ Group VI TMDs, which have been of most research interest for the synthesis of chiral 2D TMD nanomaterials, are primarily found in a trigonal prismatic geometry due to its increased thermodynamic stability. The 1T phase differs in its structure and coordination, and subsequently its electronic properties (Figure 4b).^[^ ^64^ ^]^ It belongs to the trigonal system and exhibits *AbA* stacking. The TM sites are octahedrally coordinated (Figure 4a), which leads to a partial filling of two of the three degenerate d_xy_, d_xz_, and d_yz_ orbitals. This gives rise to the metallic behavior of the 1T phase. Furthermore, the formation of Mo‐Mo clusters in the 1T phase gives the formation of a rich variety of phases (1T’, 1T’’, and 1T’’’).^[^ ^64^ ^]^ The 3R phase is less commonly reported but exhibits trigonal prismatic transition metal coordination environments similar to the 2H phase and is also semiconducting. Its crystal structure however, is rhombohedral, with three monolayers making up its unit cell, chalcogenide, and TM layers stacking in an *AbA BcB CaC* ordering (Figure 4c).^[^ ^55^ ^]^ In contrast to the other two phases, it does not have an inversion center.^[^ ^65^ ^]^ The most favorable phase for a particular TMD material is influenced by the d‐electron count of its TM. For example, Group IV TMDs with d^0^ TM centers adopt primarily octahedral coordination, but TMs in Group VI TMDs preferentially occupy trigonal prismatic sites.

### 1.3 TMDs Synthesis

TMD nanomaterials can be synthesized by a wide variety of both top‐down and bottom‐up procedures (Figure 4d). Top‐down methodology involves the cleavage of weak van der Waals bonds between TMD layers in their bulk materials, and subsequent separation or processing of the materials to achieve greater homogeneity in layer numbers or lateral sizes. The main top‐down synthetic procedures employed to produce nanoscale TMDs are chemical and electrochemical exfoliation,^[^ ^66^, ^67^ ^]^ LPE,^[^ ^68^ ^]^ and mechanical exfoliation.^[^ ^69^ ^]^

#### 1.3.1 Chemical Exfoliation

Chemical exfoliation was the earliest method employed to produce delaminated TMD nanomaterials.^[^ ^70^ ^]^ In particular, intercalation of alkali metals such as n‐butyllithium in hexane was used initially.^[^ ^71^, ^72^ ^]^ An intercalation reaction, as defined by IUPAC, is a “reaction, generally reversible, that involves the penetration of a host material by guest species without causing a major structural modification of the host,” where the guest species, intercalants, are not randomly distributed but occupy positions predetermined by the structure of the host material, for example, the interlayer spacings in TMDs.^[^ ^73^ ^]^ The process is often used in tandem with LPE or ultrasonication, which proceeds with increased efficacy due to the expansion of interlayer spacings and subsequent weakening of van der Waals forces. Electrochemical exfoliation processes have also been employed for the production of TMD nanomaterials, often using lithium or other alkali metal anodes and TMD‐coated cathodes.^[^ ^74^, ^75^ ^]^ These methodologies often benefit from the increased driving force for intercalation and gas evolution aiding in the separation of layers. Lithium ions are often favored in alkali ion methods due to their smaller atomic radius, but Na^+^ and K^+^ intercalation has also been used.^[^ ^76^ ^]^ Alkali ion chemical exfoliation methods overall suffer from a lack of scalability due to the frequent necessity of inert environments, oxygen and moisture‐free conditions, and gloveboxes for manipulations. To mitigate some of these obstacles organic cations, including quaternary ammonium cations such as cetrimonium bromide (CTAB) and tetraheptylammonium bromide (THAB), have also been widely utilized for intercalation.^[^ ^77^, ^78^ ^]^ In particular, the use of sterically bulky ions such as THAB can reduce the extent of electron injection into the TMD structure, which is often seen to bring about a phase transition from the semiconducting 2H phase to the metallic 1T phase.^[^ ^79^, ^80^ ^]^

#### 1.3.2 LPE

One of the most utilized top‐down procedures for the production of TMD nanomaterials has been LPE. LPE of TMD materials involves the ultrasonication or high shear mixing of bulk TMDs in solvents whose surface energies have been carefully selected to encourage the breaking apart of layers, or in surfactant and polymer solutions, which play a similar role. This was carried out for TMD nanomaterials firstly in a large array of organic solvents such as alkyl pyrrolidone solvents and alcohols,^[^ ^59^ ^]^ and soon after in aqueous surfactant solutions.^[^ ^81^ ^]^ Liquid‐phase shear exfoliation of TMDs was also carried out with both industrial shear mixers^[^ ^82^, ^83^ ^]^ and kitchen blenders.^[^ ^83^ ^]^ LPE leads to very processable dispersions which can be drop cast,^[^ ^84^ ^]^ used for inkjet printing,^[^ ^85^ ^]^ and spin coated.^[^ ^79^ ^]^ A post‐exfoliation size selection step is often employed to narrow the wide distribution in nanosheet lateral size and thickness often produced by this method. LPE suffers the drawback of producing nanosheets with comparatively small lateral dimensions due to sonication‐induced scission of larger sheets with prolonged sonication times,^[^ ^86^ ^]^ and this adds to the lack of control of size of sheets produced. This can be remedied somewhat by a controlled post‐LPE centrifugation approach where rotation speed is tuned to select fractions of particular lateral size regimes.^[^ ^86^ ^]^ Larger and denser particles sediment faster than smaller and less dense ones, allowing the separation of larger particles into a pellet while smaller particles remain dispersed in solution. Another size selection approach, density gradient centrifugation, allows for thickness separation of TMD nanosheet dispersions.^[^ ^87^ ^]^ Liquid cascade centrifugation is an alternative methodology to both homogeneous and density gradient centrifugation, where multiple iterations of centrifugation allow for the collection of pellets of particular size by the previous removal of larger particles and then selective crashing down of the largest of the remaining particles in dispersion and redispersion of this pellet with ultrasonication.^[^ ^88^ ^]^

#### 1.3.3 Hot Injection Synthesis

Hot injection synthesis has been employed to produce a vast library of nanomaterials including quantum dots and 2D nanomaterials. The technique involves the injection of a solution of precursor(s) into a hot solution of remaining precursor(s), leading to a rapid nucleation event and subsequent Ostwald ripening. The technique was pioneered by Bawendi et al.^[^ ^89^ ^]^ for the synthesis of CdSe colloidal nanocrystals.^[^ ^89^ ^]^ The subsequent controlled growth allows for good tunability in size and shape. One of the advantages of hot injection synthesis of TMD nanomaterials is the wide range of morphologies and shapes that can be achieved: nanoflowers, nanorods, nanosheets, and quantum dots have been produced for example.^[^ ^14^ ^]^

Single and multilayer quantum dots of WSe_2_ have been produced via hot injection synthesis using tungsten hexacarbonyl as the W precursor, PhSe_2_ as the selenium source, and TOPO as the coordinating ligand to control nanoparticle growth.^[^ ^90^ ^]^ The produced multilayer WSe_2_ QDs were then exfoliated to produce single monolayer QDs. TMD nanosheets have also been prepared via hot injection. These syntheses often employ capping ligands like oleylamine and oleic acid, and careful selection of the binding energy of these capping ligands has been used to tune the number of layers in the nanosheets.^[^ ^91^ ^]^

#### 1.3.4 Hydro/Solvo‐Thermal Synthesis

Hydrothermal and more general solvothermal syntheses have been employed since the late 1990s to produce nanocrystalline TMD materials.^[^ ^92^ ^]^ Solvothermal synthesis involves subjecting solutions of precursors to high temperature and pressure regimes, generally with the use of an autoclave or similar closed reaction vessel. Gas‐phase syntheses of MoS_2_ nested fullerenes from H_2_S and MoO_3_ in a reducing atmosphere at temperatures up to 900 °C had been carried out prior,^[^ ^93^ ^]^ but the adoption of solvothermal methodology allowed for a decrease to temperatures of a few hundred degrees and microwave heating proved capable to further reduce the reaction time to a few minutes.^[^ ^94^ ^]^ After the first evidence on the production of 1T‐MoS_2_ by Liu et al.^[^ ^95^ ^]^ hydrothermal synthesis has been utilized for the production of metallic TMD nanomaterials. MoS_2_ nanoflowers for lithium‐ion capacitors were produced via a hydrothermal synthesis using ammonium heptamolybdate tetrahydrate as a Mo source and thiourea as a sulfur source.^[^ ^96^ ^]^

#### 1.3.5 Coprecipitation

Coprecipitation reactions have been used to produce TMD quantum dots via a simple bottom‐up procedure at room temperature.^[^ ^97^, ^98^ ^]^ An example is the reaction between sodium sulfide and molybdenum pentachloride or molybdenum trioxide in alkaline solutions of bovine serum albumin, which was successfully applied for the production of MoS_2_ QDs. Stabilization with different biomolecules was observed to be critical to control the particle size and stability.^[^ ^98^ ^]^ This simple approach showed large compatibility with a broad range of stoichiometries to achieve control over the composition of defects. Moreover, the production of other TMDs with similar strategies by simply changing the molecular precursors has been verified.^[^ ^98^ ^]^

#### 1.3.6 Vapor‐Phase Synthesis

Synthetic approaches like CVD, and related procedures like physical vapor deposition (PVD) and vapor‐phase transport (VPT) are extensively employed approaches for the production of TMD nanostructures with high control of the size, morphology, and orientation according to conditions used in the crystal growth. These synthetic approaches have been discussed in great detail by other reviews on the topic and are only briefly mentioned in this section.^[^ ^99^, ^100^ ^]^ Vapor‐phase syntheses were initially known to produce inorganic fullerenes nanoparticles and rods,^[^ ^101^, ^102^ ^]^ however, due to several efforts spent on the optimization of the synthetic conditions, these strategies were successfully adapted to favor the growth of 2D layers and 1D nanotubes.^[^ ^99^, ^103^ ^]^ The first examples of CVD growth of 2D MoS_2_ crystals were reported in 2012, when Lee et al.^[^ ^104^ ^]^ presented the first observation of the production of large‐area 2D films via CVD growth using MoO_3_ and sulfur powder as precursors. In the same year, Shi et al.^[^ ^105^ ^]^ reported the growth of MoS_2_ flakes on a substrate coated with graphene using ammonium thiomolybdate as a single‐source precursor forming a 2D hybrid heterostructure. These approaches have been successfully adapted for the growth of different 2D TMD layers.^[^ ^106^, ^107^ ^]^ Though vapor‐phase techniques can give valuable control of thickness and layer numbers in TMDs, they can suffer from a lack of controllability in nanostructure morphology and growth mechanism, even within one synthesis. Prepared nanostructure samples often contain combinations of hexagonal and triangular and other morphologies, of varying sizes, with both layer‐by‐layer and screw dislocation directed growth (SDD) (discussed later).^[^ ^108^ ^]^ The observed morphologies depend strongly on substrate and precursor separation distance, substrate orientation, and local precursor concentrations and temperatures.^[^ ^109^, ^110^ ^]^ However, many efforts have been targeted toward large‐domain epitaxial growth, for example, on the wafer scale suitable for electronic applications. There are several different approaches to this growth, all with significant challenges. In particular, the need for multiple elements makes single nucleation epitaxial growth more difficult than monoelemental layered materials such as graphene.^[^ ^111^ ^]^ Multinucleation strategies require careful substrate selection and preparation, as well as fine‐tuning of precursor parameters such as precursor flux to avoid the formation of grain boundaries and to produce large‐scale uniform films. Despite these intrinsic complications, several examples of large‐sized high‐quality epitaxially grown TMDs have been reported. One example, from Yu et al.,^[^ ^112^ ^]^ reports the epitaxial growth of eight inch wafer‐scale highly oriented monolayer MoS_2_ on sapphire. Other developments in scalability of vapor‐phase synthesis of TMDs include the development of multi‐wafer deposition techniques. Kang et al.,^[^ ^113^ ^]^ for example, carried out uniform WSe_2_ deposition onto three wafers in tandem, using a travelling flow‐type reactor.

## 2 Chirality in TMDs Nanostructures Produced via Solution‐Phase Syntheses

**Figure 5:**
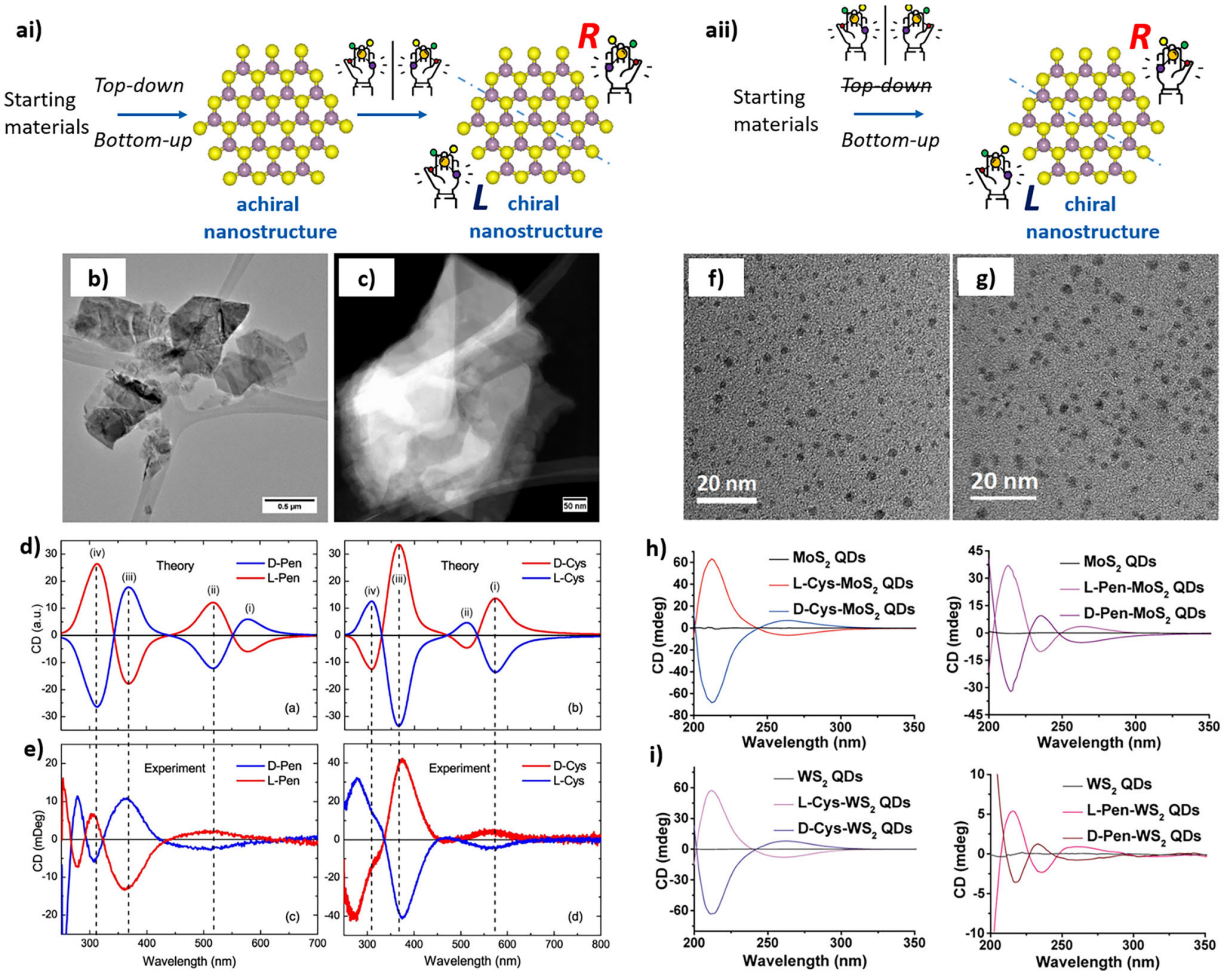
Synthetic scheme of the typical strategies adopted for the production of chiral inorganic nanomaterials via solution‐phase approach, multi‐step method (ai), and single‐step method (aii). b) TEM micrograph of MoS_2_ flakes produced in the presence of L‐cysteine. c) Scanning TEM micrograph of MoS_2_ flakes produced in the presence of D‐penicillamine. Simulated (d) and experimental (e) CD spectra of chiral MoS_2_ flakes produced by exfoliation in the presence of penicillamine (left) and cysteine (right). TEM micrograph of MoS_2_ (f) and WS_2_ (g) QDs. CD spectra of MoS_2_ (h) and WS_2_ (i) QDs functionalized by cysteine (left) and penicillamine (right). (b–e) Adapted with permission from Purcell‐Milton et al.,^[^ ^117^ ^]^ copyright 2018 American Chemical Society (f–i) adapted with permission from Zhang et al.^[^ ^118^ ^]^ copiright 2018 American Chemical Society.

As previously mentioned, solution‐phase syntheses have been extensively investigated for the production of chiral inorganic nanostructures. The advantage of these approaches is the compatibility with chiral ligands, which can be used to control the breaking of mirror symmetry, thus achieving enantioselectivity in the production of the chiral nanostructure. For convenience, chiral ligands are often chosen from the chiral pool and typically are polar molecules like amino acids, and thus the synthesis must be designed according to the compatibility with the chiral ligand. In particular, two main synthetic strategies are typically adopted: multi‐step synthesis (Figure 5ai) where the achiral colloidal nanocrystal dispersion is produced (either by top‐down or bottom‐up approaches), and then the chirality is introduced in a second step by functionalizing the nanocrystal surface with chiral ligands. By contrast, the single‐step synthesis (Figure 5aii) relies upon combining nanocrystal nucleation and growth in the presence of the chiral ligand (typically compatible only with bottom‐up synthesis), achieving at the same time nanocrystal formation and symmetry breaking. These strategies have been implemented for the production of chiral TMDs, and some details on works that explore the chirality in solution‐phase TMDs are reported in Table 1. Chiral hybrid superlattices are an exception to the typical chirality observed for TMD nanomaterials produced in colloidal solutions. These hybrid systems are produced by the intercalation of chiral molecules in the layer spacing of bulk TMD crystals and present some similarities with other chiral TMDs characterized by ligand‐induced chirality.^[^ ^114^ ^]^ Recent studies on TMD‐based chiral hybrid superlattices revealed promising chirality‐induced spin selectivity properties and unconventional superconductivity, showing potential as highly tunable topological quantum materials, for these reasons these systems are included in this paragraph.^[^ ^114^, ^115^, ^116^ ^]^

**Table 1:** Details regarding the solution‐phase synthesis of chiral TMDs.

TMDs	Chiral ligand	Chiral Origin^*^	Synthesis^**^	TMD Precursor	Morphology	Application	Ref.
MoS_2_	Cysteine Penicillamine	LI/M	i LPE/post‐synthetic modification	MoS_2_	Flakes	‐	[117]
	Cysteine Penicillamine	LI	i LPE/post‐synthetic modification	MoS_2_	QDs	Enantioselective catalysis	[118]
	MBA	LI	ii Hydrothermal	(NH_4_)_2_MoS_4_	Flower‐like NPs	CISS/OER Spintronics	[119]
	Cysteine	LI	i Hydrothermal/post‐synthetic modification	(NH_4_)_6_Mo_7_O_24_ CoCl_2_	Flower‐like NPs	Nanomedicine	[120]
	Penicillamine	LI	i LPE/post‐synthetic modification	MoS_2_	QDs	Nanomedicine	[121]
	Cysteine	LI/M	ii Coprecipitation	MoO_3_	QDs	‐	[122]
	Tartaric acid	M	ii Hydrothermal	(NH_4_)_6_Mo_7_O_24_	Twisted nanosheets	‐	[123, 124]
MoSe_2_	Penicillamine	LI	Ii Coprecipitation	MoCl_5_	QDs	Nanomedicine	[125]
WS_2_	Cysteine Penicillamine	LI	i LPE/post‐synthetic modification	WS_2_	QDs	Enantioselective catalysis	[118]
WSe2	Cysteine Penicillamine	LI	i LPE/post‐synthetic modification	WSe_2_	QDs	Nanomedicine	[126]
TaS_2_	MBA	LI	intercalation	TaS_2_	Chiral superlattice	CISS Spintronics	[114, 115]
TiS_2_	MBA	LI	intercalation	TiS_2_	Chiral superlattice	CISS Spintronics	[114]
	PEA	LI	intercalation	TiS	Chiral superlattice	CISS/OER Spintronics	[116]

* Origin of chirality: ligand induced (LI), chiral morphology (M).

** Synthesis: two‐step (i) and single‐step (ii) approaches, LPE.

The first observation of chirality in TMDs was reported by Purcell‐Milton et al.^[^ ^117^ ^]^ using chiral thiol ligands like cysteine and penicillamine as chiral inductors in MoS_2_ flakes. The chiral MoS_2_ flakes were produced by a top‐down two‐step approach based on the LPE of bulk MoS_2_ followed by a second sonication step in the presence of the chiral ligand. In this way, colloidal dispersions of MoS_2_ flakes with a large aspect ratio were produced. Atomic force microscopy (AFM) analysis revealed a thickness of 3–4 nm (4–6 S‐Mo‐S monolayers) and 5–9 nm (7–14 S‐Mo‐S monolayers) for the flakes produced in the presence of penicillamine and nonfunctionalized, respectively, while TEM analysis showed a lateral size ranging from hundreds of nanometers to 2–3 µm (Figure 5b,c). The CD characterization of the chiral MoS_2_ flakes showed intense CD features (Figure 5d,e) located in the UV region and a broad, weaker transition in the visible range between 450 and 700 nm. In particular, a strong band located between 350 and 400 nm was observed for both cysteine and penicillamine functionalized MoS_2_ flakes. The authors ascribed the chiroptical activity to the effect of the chiral ligand on the splitting of the excitonic transition, analogous to what had been previously reported by Ben‐Moshe et al.^[^ ^33^ ^]^ for CdSe and CdS QDs, where the narrow excitonic transition of the QDs was observed as two distinctive CD bands with opposite signs producing a Cotton‐like effect. This behavior was associated with the splitting of the fundamental transition into two transitions excited preferentially by oppositely circularly polarized light. Interestingly, other amino acids were tested to introduce the chiroptical properties in MoS_2_ flakes without showing any significant chiroptical activity besides the contribution arising from the ligand itself. In order to explain the CD intensity, which was found to be higher than what was expected for a purely ligand‐induced‐based system,^[^ ^2^, ^17^ ^]^ the authors performed computational simulations to investigate the effect of structural distortion induced by the chiral ligand on the MoS_2_ flakes’ morphology. The chiral MoS_2_ flakes were approximated as chirally distorted plain nanoplates, and the CD signal was simulated by Rosenfeld's approximation, giving good agreement with the empirical results (Figure 5d,e).

**Figure 6:**
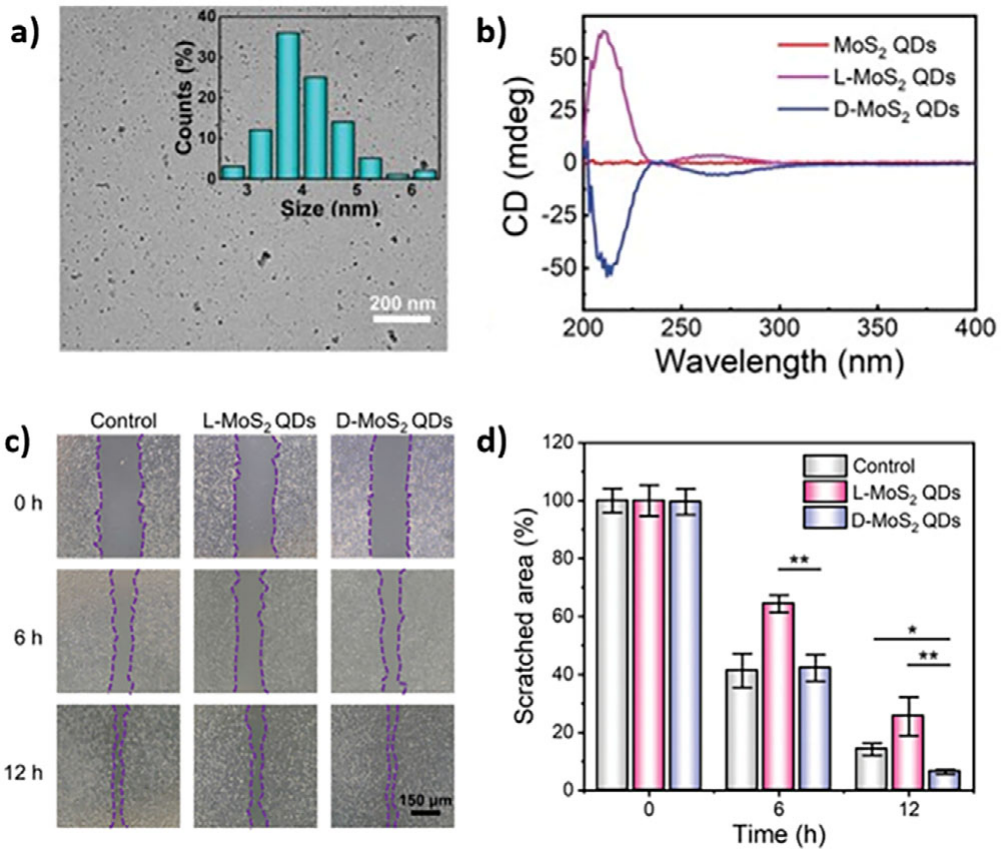
a) TEM micrograph of MoS_2_ QDs functionalized with L‐penicillamine and size distribution histogram (inset). b) CD spectra of MoS_2_ QDs before and after functionalization with L‐ and D‐penicillamine. c,d) Investigation of the angiogenic activity of chiral MoS_2_ QDs using scratched HUVECs treated at different times. Figure adapted with the permission of Liang et al.^[^ ^121^ ^]^

In a similar two‐step top‐down approach, Zhang et al.^[^ ^118^ ^]^ produced chiral MoS_2_ and WS_2_ quantum dots showing interesting peroxidase‐like activity.^[^ ^127^, ^128^ ^]^ In this case, after the sonication of the bulk MoS_2_ and WS_2_ powders to produce a TMD colloidal dispersion, the size selection step was optimized to collect the smaller QD‐type nanoparticles instead of larger flakes.^[^ ^86^, ^129^ ^]^ TEM analysis (Figure 5f,g) revealed the presence of nanoparticles with average sizes of 2.03 ± 0.48 nm and 2.00 ± 0.59 nm for MoS_2_ and WS_2,_ respectively. Interestingly, these TMD QDs showed excitation‐dependent photoluminescence properties. The authors tuned the emission of MoS_2_ QDs between 436 and 529 nm by changing the excitation from 350 to 450 nm. Similarly, the emission peak of WS_2_ QDs was varied from 437 to 484 nm by altering the excitation in the 360 to 410 nm range. The TMDs QDs were then functionalized with chiral ligands (cysteine and penicillamine). Cysteine‐functionalized MoS_2_ and WS_2_ QDs both showed CD spectra dominated by two main transitions centered at 212 and 265 nm with opposite signs (Figure 5 h,i). Penicillamine‐functionalized samples showed the presence of peaks at 235 and 264 nm. The authors associated this feature with the Cotton effect expected from the dipolar interaction between the QD exciton and molecular orbitals of the chiral ligand. Interestingly, chiral TMDs QDs showed potential as enantioselective catalysts. The peroxidase‐like activity of the chiral MoS_2_ QDs was investigated by monitoring the oxidation of TMB (3,3′,5,5′‐tetramethylbenzidine) with H_2_O_2_ showing negligible peroxidase activity for both MoS_2_ and cysteine‐modified chiral MoS_2_ QDs, however, a modification with copper (II) greatly increased their catalytic activity.^[^ ^130^, ^131^ ^]^ The enantioselective peroxidase‐like activity of these systems was then exploited for the oxidation of L‐ and D‐tyrosine, giving quite distinctive values for Michaelis‐Menten constants and maximum reaction rates. A superior affinity for L‐tyrosine was observed for L‐cysteine‐functionalized MoS_2_ QDs, and oppositely, the MoS_2_ QDs functionalized with D‐cysteine showed higher affinity for D‐tyrosine. These conclusions based on the catalytic activity were further validated by the investigation of preferential binding of tyrosine enantiomers using a quartz crystal microbalance. This first report on the enantioselective nanozyme behavior of TMD QDs^[^ ^118^ ^]^ inspired several other works focusing on their interaction with biological systems.^[^ ^121^, ^125^, ^126^ ^]^ Among these, Liang et al.^[^ ^121^ ^]^ investigated the chirality‐dependent angiogenic activity of chiral MoS_2_ QDs functionalized with penicillamine. Even in this case, the chiral TMDs QDs were prepared by a two‐step top‐down synthesis using penicillamine as the chiral ligand. However, in contrast with the procedure already discussed, Liang et al.^[^ ^121^ ^]^ relied on a solvothermal step to produce the MoS_2_ QDs from the bulk powder.^[^ ^132^ ^]^ In this step, the bulk MoS_2_ powder was dispersed in an alkaline ethanol solution and treated in a Teflon‐lined, stainless‐steel autoclave at 180 °C for 12 hours. The TMD dots were then collected in the supernatant after centrifugation and finally purified by dialysis. The chirality was induced by a mild functionalization of the MoS_2_ QDs with penicillamine by mixing a solution of penicillamine, at pH 12, with the QD dispersion in ethanol and stirring overnight at room temperature‐exploiting the particular affinity of thiols for sulfur vacancies on the MoS_2_ QDs’ surface.^[^ ^133^, ^134^ ^]^ TEM analysis revealed the presence of nanoparticles with an average size of 4.1 ± 0.7 nm and 4.3 ± 0.9 nm for the sample functionalized by L‐ (Figure 6a) and D‐penicillamine, respectively. After functionalization, the MoS_2_ QDs showed CD spectra with a strong peak located at 212 and a second weaker peak at around 265 nm (Figure 6b). Interestingly, in‐vitro investigation of the angiogenesis activity on a scratched assay (Figure 6c,d) revealed a preferential effect of D‐Pen functionalized MoS_2_ QDs to induce the proliferation and migration of HUVECs cells. Further investigation on the growth of HUVAC cells on Matrigel showed an increase in the formation of a capillary‐like network for the group treated with MoS_2_ QDs functionalized with D‐penicillamine. The internalization of MoS_2_ QDs by HUVAC cells was investigated by confocal imaging and ICP‐MS, revealing an enantioselective uptake for the D‐Pen‐functionalized MoS_2_ QDs which can be related to preferential interaction with the cell membrane. These observations were further supported by the in vivo studies of the wound‐healing performance of chiral MoS_2_ QDs on mouse models with full‐thickness cutaneous defects, showing an accelerated re‐epithelization and collagen deposition in the presence of MoS_2_ QDs functionalized with D‐penicillamine.

**Figure 7:**
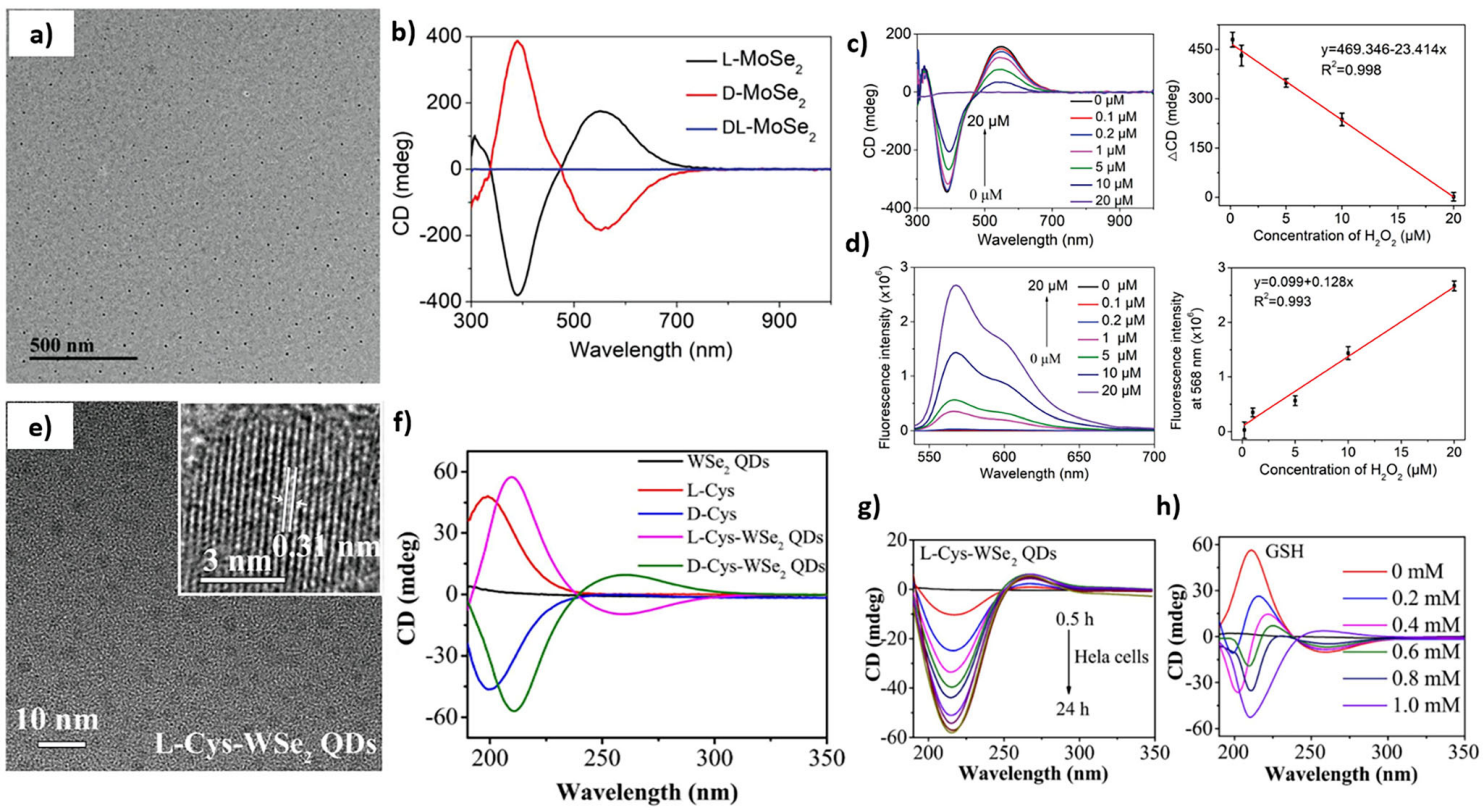
TEM micrograph (a) and CD spectra (b) of chiral MoSe_2_ QDs produced in the presence of penicillamine. Analysis of the chiral MoSe_2_ QDs response to hydrogen peroxide addition in different concentrations using CD (c) and PL (d). TEM (e) micrograph of chiral WSe_2_ QDs obtained by functionalization with L‐cysteine. f) CD spectra of chiral WSe_2_ QDs functionalized by cysteine. g) Effect of the incubation time in HeLa cells on the CD spectra of WSe_2_ QDs functionalized by L‐cysteine and (h) CD spectra of the reaction of chiral WSe_2_ QDs with glutathione in different concentrations. (a‐d) Adapted with permission from Cao et al.,^[^ ^125^ ^]^ and (e‐g) adapted with permission from Yang et al.^[^ ^126^ ^]^

Another active area of research regarding the application of chiral TMDs QDs related to nanomedicine has been focused on their performance as nanosensors for the intracellular detection of reactive oxygen species (ROS). In this context, it is worth mentioning the work of Cao et al.^[^ ^125^ ^]^ on chiral MoSe_2_ QDs and the work of Yang et al.^[^ ^126^ ^]^ covering WSe_2_ QDs, where CD and PL signals were used to monitor intracellular ROS levels in both in vitro and in vivo experiments. In the former case,^[^ ^125^ ^]^ chiral MoSe_2_ QDs were produced by a two‐step one‐pot process using SeO_2_ solubilized in sodium borohydride and MoCl_5_ to form MoSe_2_ QDs, then, after cooling, penicillamine was added as a chiral ligand to functionalize the QDs’ surface. The product's morphology was characterized by TEM (Figure 7a) which showed the presence of nanoparticles with an average diameter of 3.4 ± 1.2 nm. CD spectroscopy of QDs produced in the presence of L‐penicillamine showed a strong negative band located at 390 nm and a second, positive, weaker band at 550 nm (Figure 7b). Further functionalization with cyanine 3 allowed the introduction of a photoluminescent probe on the QDs’ surface. In particular, in this system, the photoluminescence of cyanine 3 was quenched when the fluorophore was bonded to the QDs, but it could be restored by the ROS activity. The authors demonstrated that both CD and PL signals were sensitive to the ROS content (Figure 7c,d), These properties were related to the oxidation of MoSe_2_ by hydrogen peroxide, which led to the formation of achiral MoO_3_ nanoparticles, revealing a promising application of chiral TMDs in nanomedicine. Yang et al.^[^ ^126^ ^]^ examined WSe_2_ QDs produced by a two‐step synthesis based on firstly a top‐down solvent exfoliation process to produce small QDs of a few nanometers (Figure 7e) from the bulk powder, followed by functionalization with cysteine and penicillamine to introduce ligand‐induced chirality. CD investigations of WSe_2_ QDs functionalized by cysteine (Figure 7f) revealed spectra dominated by two main bands with opposite signs located in the ultraviolet region. The most intense band was observed at around 214 nm and a second, weaker band at 262 nm. Interestingly, the CD signal of WSe_2_ QDs was observed to be invertible after incubation in HeLa cells for an extended time (Figure 7g). This process was related to the intracellular transformation of the surface cysteine to glutathione by the enzymatic action of the glutamate‐cysteine ligase and glutathione synthetase.^[^ ^135^ ^]^ Besides being a potential sensor for ROS, these TMD QDs were shown to be efficient in mimicking glutathione peroxidase activity with important implications in the regulation of intracellular ROS levels. XPS and FTIR analyses revealed that this process proceeded by the formation of Se ═ O bonds due to the reaction with hydrogen peroxide, which could then be reduced at the expense of glutathione. Moreover, enantioselective activity and a faster initial reaction rate were observed for WSe_2_ QDs functionalized by L‐cysteine.

An interesting example of a single‐step bottom‐up approach for the production of MoS_2_ QDs was reported by Luo et al.^[^ ^122^ ^]^ using MoO_3_ as a molybdenum precursor, NaSH solution as a source of sulfide anions, L‐ascorbic acid as a reducing agent, and cysteine as a chiral ligand. TEM characterization revealed the presence of nanoparticles with a size of around 8 nm, and HR‐TEM analysis showed fringes with a d‐spacing compatible with (103) planes of MoS_2_. CD characterization revealed spectra characterized by two main transitions located at approximately 375 and 400 nm, and the authors associated the CD signal above 400 to the intrinsic semiconductor properties of MoS_2_. Due to the similarity in the chiroptical properties, the authors suggested a similar origin of chirality to the MoS_2_ flakes reported by Purcell‐Milton et al.^[^ ^117^ ^]^ thus, the observed chirality being attributed to a distortion of the nanodot's morphology introduced by the chiral ligand.

**Figure 8:**
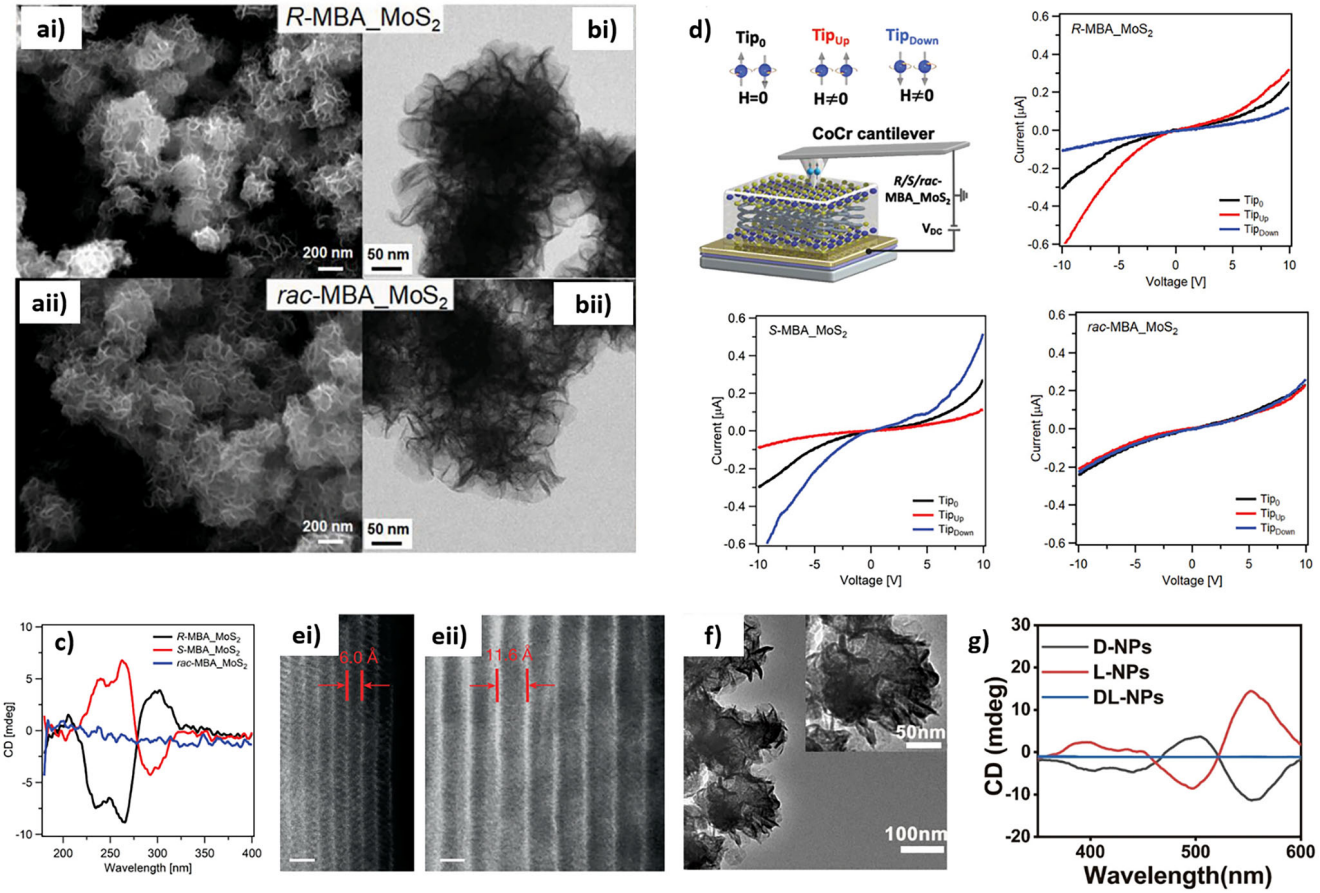
SEM (a) and TEM (b) characterization of hybrid chiral MoS_2_ produced in the presence of R‐MBA (ai, bi) and rac‐MBA (aii, bii). c) CD spectra of the hybrid chiral MoS_2_. d) Investigation of the chirality‐induced spin selectivity of hybrid chiral MoS_2_ produced in the presence of R, S, and *Rac*‐MBA using spin‐polarized c‐AFM. TEM micrographs of TMDs superlattices before (ei) and after (eii) the MBA intercalation. TEM micrograph (f) and CD spectra (g) of chiral MoS_2_/CoS_2_ heterostructure. (a‐d) Adapted from Bian et al.,^[^ ^119^ ^]^ (e) adapted with permission from Qian et al.^[^ ^114^ ^]^ and (f,g) adapted with permission from Zhang et al.^[^ ^120^ ^]^

The production of chiral TMDs nanocrystals with complex 3D structures was achieved by hydrothermal reaction.^[^ ^119^, ^120^, ^123^ ^]^ Hydrothermal and solvothermal processes are based on reactions at temperatures beyond the boiling point of the solvent, using sealed vessels to produce autogenic pressure mainly according to temperature and filling fraction. These syntheses permit high control of morphology, chemical composition, and crystallographic structure.^[^ ^136^, ^137^, ^138^, ^139^ ^]^ In particular, hydrothermal synthesis has been implemented for the production of metallic 1T‐MoS_2_ at large scales.^[^ ^13^, ^95^ ^]^ The synthesis of a hybrid chiral MoS_2_ was reported by Bian et al.^[^ ^119^ ^]^ using a single‐step hydrothermal process in the presence of (NH_4_)_2_MoS_4_ as a single‐source precursor of molybdenum and sulfur and methyl benzylamine (MBA) as a chiral inducer. SEM and TEM analysis revealed the flower‐like MoS_2_ nanocrystals formed by aggregated thin nanosheets, with an average diameter of around 200 nm (Figure 8a,b). The CD analysis in thin films (Figure 8c) revealed peaks at 206, 240, 263, and 298 nm. The chirality‐induced spin selectivity (CISS) properties of the hybrid chiral MoS_2_ were investigated by spin‐polarized conductive atomic force microscopy (c‐AFM) measurements (Figure 8d) and a spin polarization of +71 and ‐75% at ‐10 V bias was observed for the MoS_2_ nanoflowers produced in the presence R and S‐MBA, respectively, disclosing potential application of chiral hybrid MoS_2_ in spintronics. The material was further investigated as a catalyst for oxygen evolution reaction (OER) using a glassy‐carbon electrode modified with hybrid chiral MoS_2_. The analysis of the Tafel plots revealed slopes of 506 mV dec^−1^ observed for the racemate and > 428 mV dec^−1^ and > 395 mV dec^−1^ observed for R‐ and S‐MBA, respectively, which the authors revealed a faster OER kinetic for chiral hybrid MoS_2_ with respect to the achiral racemate. Measurements on the production of hydrogen peroxide under OER conditions revealed a suppressed formation of the peroxide for the chiral MoS_2_ nanoflowers. The authors ascribed this observation to the favored formation of hydroxyl radicals aligned in parallel orientation due to the CISS effect, which would promote the production of triplet oxygen with respect to the spin‐forbidden coupling, which leads to the formation of hydrogen peroxide. Similarly, other chiral hybrid systems formed by the intercalation of chiral organic molecules in TMDs crystals forming chiral hybrid superlattices proved a reliable strategy for the production of advanced materials with potential application in spintronics. In their seminal work, Qian et al.^[^ ^114^ ^]^ explored 2H and 1T‐TaS_2_ and 1T‐TiS_2_ intercalated with different chiral ligands, including α‐methylbenzylamine (MBA), to create chiral molecular intercalated superlattices. TEM analysis of the interlayer distance clearly revealed an expansion following the intercalation process (Figure 8ei,ii). This simple approach allowed the production of robust systems which revealed high spin polarization up to around 60%.^[^ ^114^ ^]^ Interestingly, 2H‐TaS_2_ intercalated with MBA exhibited unconventional superconductivity associated with the incorporation of the organic chiral molecules.^[^ ^115^ ^]^ Using a similar strategy, Bia et al.^[^ ^116^ ^]^ observed a spin polarization around 100% using spin‐polarized c‐AFM in hybrid systems formed by metallic T‐phase TiS_2_ and 1‐phenylethylene amine produced by electrochemical intercalation. The chiroptical activity of these chiral hybrid superlattices is dominated by the contribution of the chiral ligand, which is intercalated between the inorganic layers, while the electronic transitions of the TMDs are usually chiroptically inactive. Despite the lack of a chirality transfer, these hybrid systems give rise to the formation of multiple tunneling junctions, which grant high control of the spin polarization.

Hydrothermal methods have proven to be particularly useful for tuning the material's chemical composition.^[^ ^139^ ^]^ In particular, Zhang et al.^[^ ^120^ ^]^ reported the synthesis of a mixed chiral MoS_2_/CoS_2_ heterostructure using a two‐step procedure. An initial hydrothermal reaction between ammonium molybdate, cobalt chloride, and thiourea was used to produce flower‐like nanoparticles with a diameter of around 150 nm (Figure 8e). The chirality was introduced in a second step by functionalizing the pre‐formed MoS_2_/CoS_2_ nanoflowers with cysteine. The chiral heterostructure exhibits CD activity in the ultraviolet to visible range (Figure 8f), however further analysis of the chirality is missing. The authors observed that the chiral MoS_2_/CoS_2_ nanoflowers showed promising peroxidase‐and catalase‐like activities, suggesting a potential application in the control of extracellular radicals and oxygen content. For this reason, this composite was investigated in the tumor‐associated macrophage polarization of the tumor microenvironment between M1 and M2 phenotype populations, which is known to be substantially influenced by the extracellular ROS.^[^ ^140^ ^]^ Chirality‐dependent macrophage polarization studies in vitro highlighted that D‐cysteine‐functionalized MoS_2_/CoS_2_ nanoflowers were significantly more active in the reprogramming of the M2‐like macrophage, which promotes tumor growth, into the M1‐like polytype.^[^ ^141^ ^]^ In vivo studies on mice bearing 4T1 tumors further supported that treatment with nanoparticles functionalized with D‐cysteine significantly suppressed the tumor growth, reduced tumor volume, and mass compared to the control groups.

**Figure 9:**
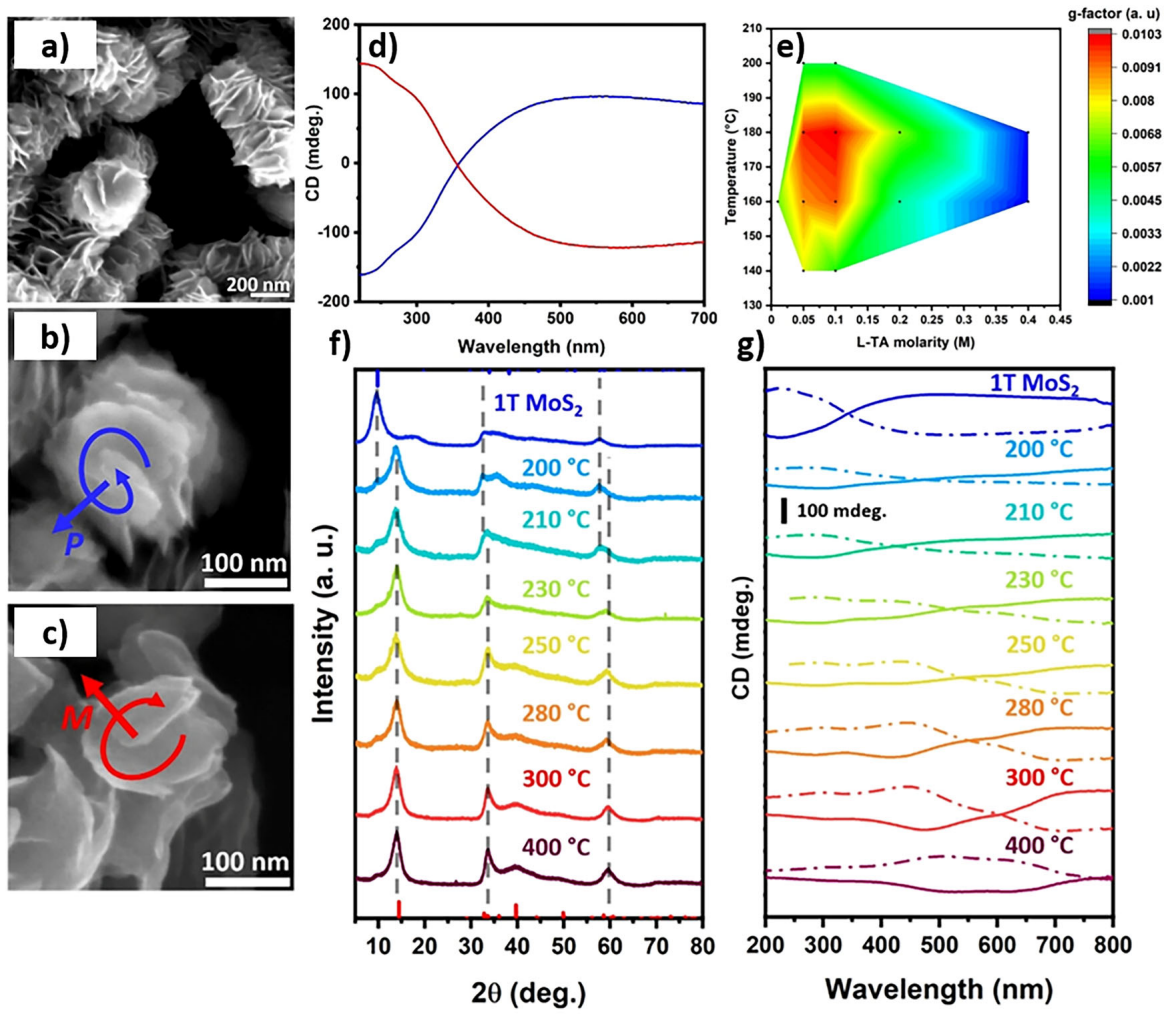
SEM micrographs of chiral MoS_2_ nanosheets produced in the presence of L (a,b) and D (c) ‐tartaric acid. CD (d) spectra of chiral MoS_2_ nanosheets produced in the presence of L (blue) and D (red) ‐tartaric acid. e) Investigation of the effect of the reaction condition on the chirality of MoS_2_ nanosheets. Effect of the thermal annealing at different temperatures on crystal structure and CD spectra of MoS_2_ produced in the presence of L (solid line) and D (dashed line) ‐tartaric acid. (a‐e) Adapted from Branzi et al.^[^ ^123^ ^]^ and (f,g) adapted from Branzi et al.^[^ ^124^ ^]^

The production of 1T‐MoS_2_ nanostructures with chiral morphology was recently reported by Branzi et al.^[^ ^123^ ^]^ using a single‐step hydrothermal synthesis. By this approach, MoS_2_ nanocrystals were produced by the reaction between ammonium molybdate and thiourea in the presence of tartaric acid as a chiral ligand to control the symmetry breaking during nanocrystal formation. SEM analysis revealed the presence of nanocrystals with a twisted morphology, which was associated with the growth of MoS_2_ nanosheets along a screw dislocation defect, producing a chiral helical structure. Nanocrystals with P and M chirality were observed when using L‐ or D‐tartaric acid during the hydrothermal synthesis (Figure 9a–c). Chiroptical characterization revealed strong CD activity covering the whole UV and visible spectrum, in alignment with the absorption properties expected for the metallic 1T‐MoS_2_ nanocrystals (Figure 9d). In particular, MoS_2_ nanocrystals produced in the presence of L‐tartaric acid showed two negative peaks in the UV range at around 230 and 290 nm and a broad absorption with a maximum at around 545 nm. The relationship between reaction conditions and chirality was investigated by tuning ligand concentration and temperature used in the hydrothermal synthesis (Figure 9e). In particular, the authors observed a critical role of the chiral ligand concentration, which was related to the variation in the nanocrystals’ morphology, giving maximum chiroptical activity for the formation of the extended twisted nanosheet structure. Finally, the authors evaluated the formation mechanism of the chiral MoS_2_ nanosheets as well as the role of the chiral ligand during the crystal growth. The chiral ligand was initially involved in the formation of chiral coordination complexes, which were deemed critical in formation of the chiral morphology, and followed by its decomposition to achiral by‐products.^[^ ^142^, ^143^, ^144^ ^]^ Furthermore, the production of chiral 2H‐MoS_2_ nanosheets exploiting a phase engineering approach was explored in a following report.^[^ ^124^ ^]^ In particular, thermal annealing in the 200–300 °C range allowed the conversion of metastable metallic 1T‐MoS_2_ to the thermodynamically stable semiconductor 2H‐MoS_2_ phase while preserving the chiral morphology. The analysis of the evolution of the crystallographic structure and chiroptical activity with the annealing temperature (Figure 9f,g) revealed the progressive evolution of the CD active bands giving rise to the formation of the characteristic excitonic features of 2H‐MoS_2_.

Solution‐phase approaches have proven successful in producing high‐quality chiral TMD nanocrystals with potential applications in sensing, nanomedicine, CISS, and others.^[^ ^119^, ^120^, ^125^, ^126^ ^]^ The superior tunability of solution‐phase syntheses allows the production of a variety of different morphologies and TMDs, as well as systems characterized by different origins of chirality. In particular, solution‐phase synthesis relies on the use of chiral ligands to control the symmetry breaking during the crystal growth or in post‐synthesis functionalization, achieving the production of systems with high stereochemical control, as proven by the observation of CD spectra for the ensemble of nanocrystals.^[^ ^145^ ^]^ Some general conclusions on the main trends in this area can be drawn. Among the different chiral ligands available to control the symmetry breaking during the nanocrystal formation, chiral thiols like penicillamine and cysteine are the most employed. Regarding the origin of chirality, most of these systems rely on ligand‐induced chirality, which is by far the most common type of chirality observed in colloidal TMDs. A particularly large amount of research deals with QD‐type morphology, likely motivated by the large interest in the application of TMDs in nanomedicine.^[^ ^146^, ^147^ ^]^

Despite the large popularity and appealing properties of TMD QDs,^[^ ^90^ ^]^ these nanosystems are still quite controversial, and particular care must be considered while assessing optical and chiroptical properties. Specifically, the co‐presence of carbon dots in the products of the synthesis of TMD QDs has been well documented, and the optical properties can often be partially or totally associated with carbon dot contaminations,^[^ ^148^ ^]^ including the characteristic excitation‐dependent photoluminescence.^[^ ^149^ ^]^ Carbon dots can be easily produced during both top‐down^[^ ^150^ ^]^ and bottom‐up^[^ ^148^ ^]^ syntheses. Hydrothermal and solvothermal approaches are well‐known procedures to synthesize carbon dots via the decomposition of organics.^[^ ^151^ ^]^ However, even less harsh conditions can cause the formation of these ubiquitous contaminations, and extended sonication of TMDs in NMP was reported to cause the formation of a substantial amount of carbon dots.^[^ ^150^ ^]^ Carbon dots can also be easily produced at room temperature by the catalytic activity of TMs.^[^ ^152^ ^]^ Moreover, carbon dots tend to retain some of the stereochemistry of the starting materials when produced in opportune conditions, and characteristic CD activity of chiral carbon dots is well‐documented.^[^ ^152^, ^153^, ^154^, ^155^, ^156^, ^157^ ^]^ These chiral carbon dots can be easily produced by the decomposition of chiral ligands like cysteine or penicillamine under thermal stress, the catalytic effect of metal ions, or extended sonication, and can give chiroptical activity very similar to TMD QDs. Thus, their potential presence should be carefully addressed, and their contribution should be verified by proper control experiments.

Among the ligands commonly adopted for the control of symmetry breaking, thiol‐based ligands are particularly appealing since they can easily passivate chalcogenide vacancy on the TMDs surface.^[^ ^133^, ^134^, ^158^ ^]^ However, thiols are also prone to involvement in other reactions, notably the oxidation to disulfide was observed to be catalyzed by 2H MoS_2_.^[^ ^159^ ^]^ Moreover, it must be remarked that contamination by coordination complexes must be carefully evaluated during the analysis of chiroptical activity via CD. The CD activity of molybdenum complexes with cysteine and tartaric acid is well documented.^[^ ^144^, ^160^, ^161^ ^]^ Several complexes can be formed in the synthetic conditions employed for the production of chiral TMDs, and their presence must be properly considered.^[^ ^162^, ^163^ ^]^ In particular, strong CD bands are observed for solutions of Mo(V) complexes with cysteine, and the Mo(V) species can be easily formed in solution by the reduction of a Mo(VI) precursor by cysteine or by the effect of other reducing agents used for the synthesis of MoS_2_.^[^ ^160^, ^163^, ^164^ ^]^ These coordination complexes can play a fundamental role in the formation of the chiral nanostructure,^[^ ^123^ ^]^ or they may remain chemically bonded to the nanoparticle surface, however, their potential contribution to the chiroptical activity must be properly addressed.

Further investigation on the origin of the chiroptical activity and the effect of the chirality on the electronic properties of TMDs must be performed. In most cases, the chiroptical activity is associated with the splitting of the main excitonic transition into two bands that are excited preferentially by circularly polarized light with different handedness, giving a sort of “’Cotton effect”’ in the CD spectra. Such a model, originally proposed by Ben‐Moshe et al.^[^ ^33^ ^]^ to describe the chiroptical activity of CdS and CdSe quantum dots stabilized by chiral molecules. However, such a model must be used carefully to describe the chiroptical properties of different systems, requiring a good comparison with the optical properties indicating the matching between the inversion of the CD signal and the maximum of the absorption peak related to the excitonic transition in question. Moreover, the chiroptical activity observed in deep electronic transitions, typically located in the UV range, is often associated with ligand‐localized states hybridized with surface states and surface local distortions induced by the chiral ligands rather than the induction of chirality in the material's electronic transitions.^[^ ^165^, ^166^, ^167^, ^168^, ^169^ ^]^

## 3 Chirality in TMD Nanostructures Produced via Vapor‐Phase Syntheses

Vapor‐phase syntheses like CVD and related approaches are largely employed for the production of high‐quality TMD nanocrystals with fine control of morphology, layer number, and crystallographic phase, which makes these approaches particularly appealing for applications in photonics, electronics, and nonlinear optics.^[^ ^100^, ^170^ ^]^ In these types of nanostructures, chirality is often observed in the morphology of spiral TMD flakes produced by SDD growth and in 1D nanotubes, which can grow with different chiral angles. Table 2 lists some relevant details on works dealing with the growth of chiral 2D TMDs nanocrystals.

**Table 2:** Details regarding the vapor‐phase synthesis of chiral TMDs spiral flakes.

TMDs	Synthesis^*^	Precursors	Morphology^**^	Growth T [°C]	Ref.
MoS_2_	CVD	MoO_3_; S	Flat spirals (T)	700	[171]
	CVD	(NH_4_)_6_Mo_7_O_24_; S	Flat spirals (T)	780	[172]
MoSe_2_	WA CVT	MoSe_2_	Flat spirals (T)	1150	[173]
MoTe_2_	CVD	(NH_4_)_6_Mo_7_O_24_; Te	Flat spirals (T)	690	[174]
WS_2_	CVD	WO_3_; S	Flat spirals (T/H)	900	[175]
	WA CVT	WS_2_	Supertwisted spirals	≈ 1000	[176]
	WA CVT/H_2_O_2_ etching	WS_2_	Etched spirals (T/H/TT)	≈ 1000	[177]
	WA CVD	WS_2_	Supertwisted spirals	700–800	[178]
WSe_2_	SA CVD	WO_3_; S; Se	Flat spirals (T/H)	875–900	[179]
	CVD	WO_3_; Se	Flat spirals (T/H/TT)	880	[180]
	WA APCVT	WSe_2_	Supertwisted spirals	≈ 1000	[176]
	CVD/H_2_O_2_ etching	WO_3_; Se	Etched spiral (T/H/TT)	880	[177]

* Synthesis: CVD, water vapor‐assisted chemical vapor transport (WA CVT), sulfur‐assisted chemical vapor deposition (SA CVD).

** Morphology: triangular (T), truncated triangular (TT) and hexagonal (H).

**Figure 10:**
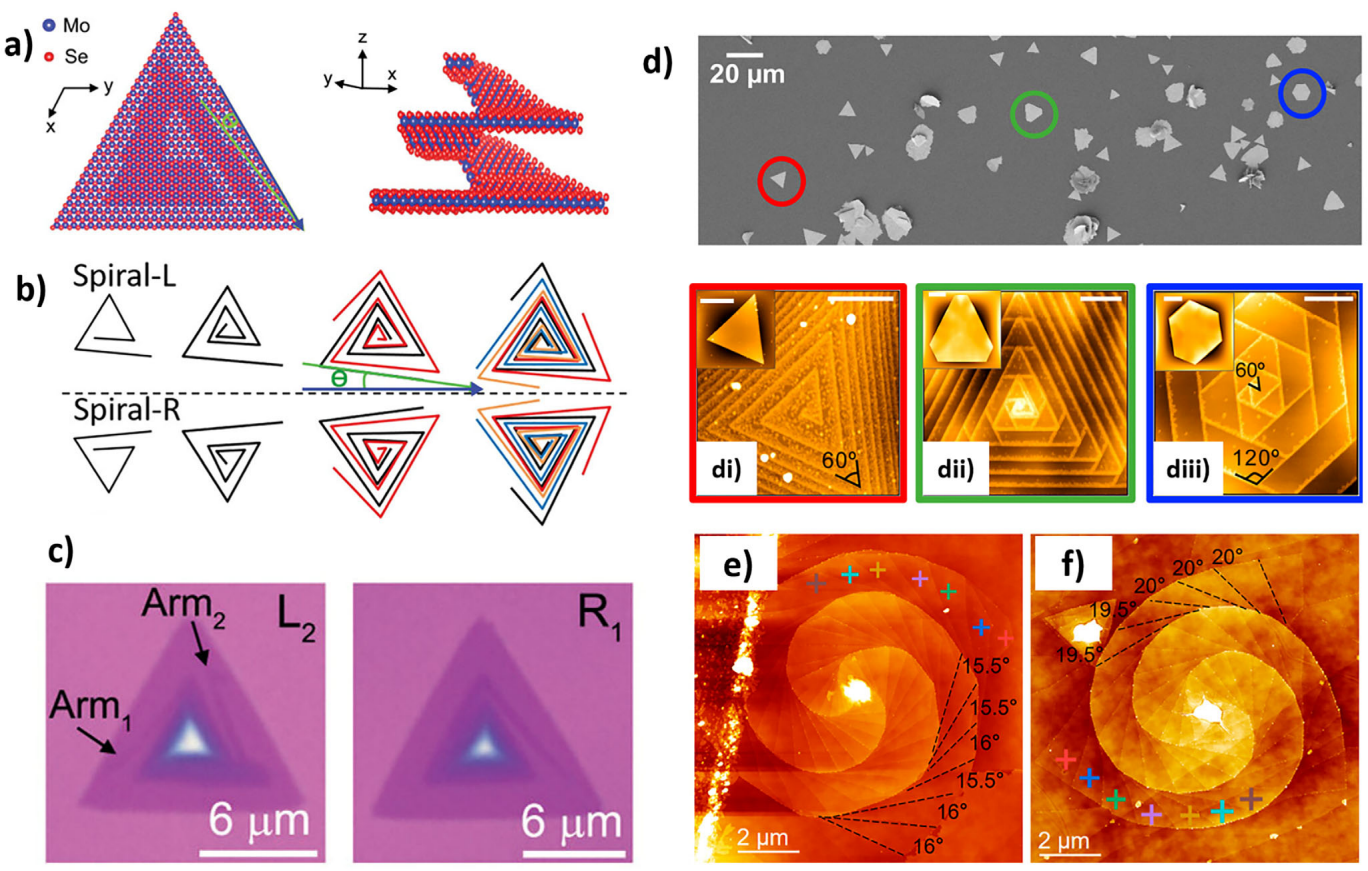
a) Atomistic model of the structure of few layer spiral MoSe_2_ flake. b) scheme representing spiral flakes characterized by different chirality and number of arms. c) Optical microscopy image of L‐(Left) and R (right) spiral MoSe_2_ flakes. d) SEM and AFM images of WSe_2_ nanoplates showing the presence of flakes with different morphologies. e) AFM images of flakes with different morphologies, T (di), TT (dii) and H (diii). e,f) AFM images of WS_2_ supertwisted spirals with different twist angles. a‐c) Adapted with permission from Wang et al.,^[^ ^173^ ^]^ (d, e) adapted from Shearer et al.,^[^ ^180^ ^]^ (f,g) adapted with permission from Tong et al.^[^ ^178^ ^]^

**Figure 11:**
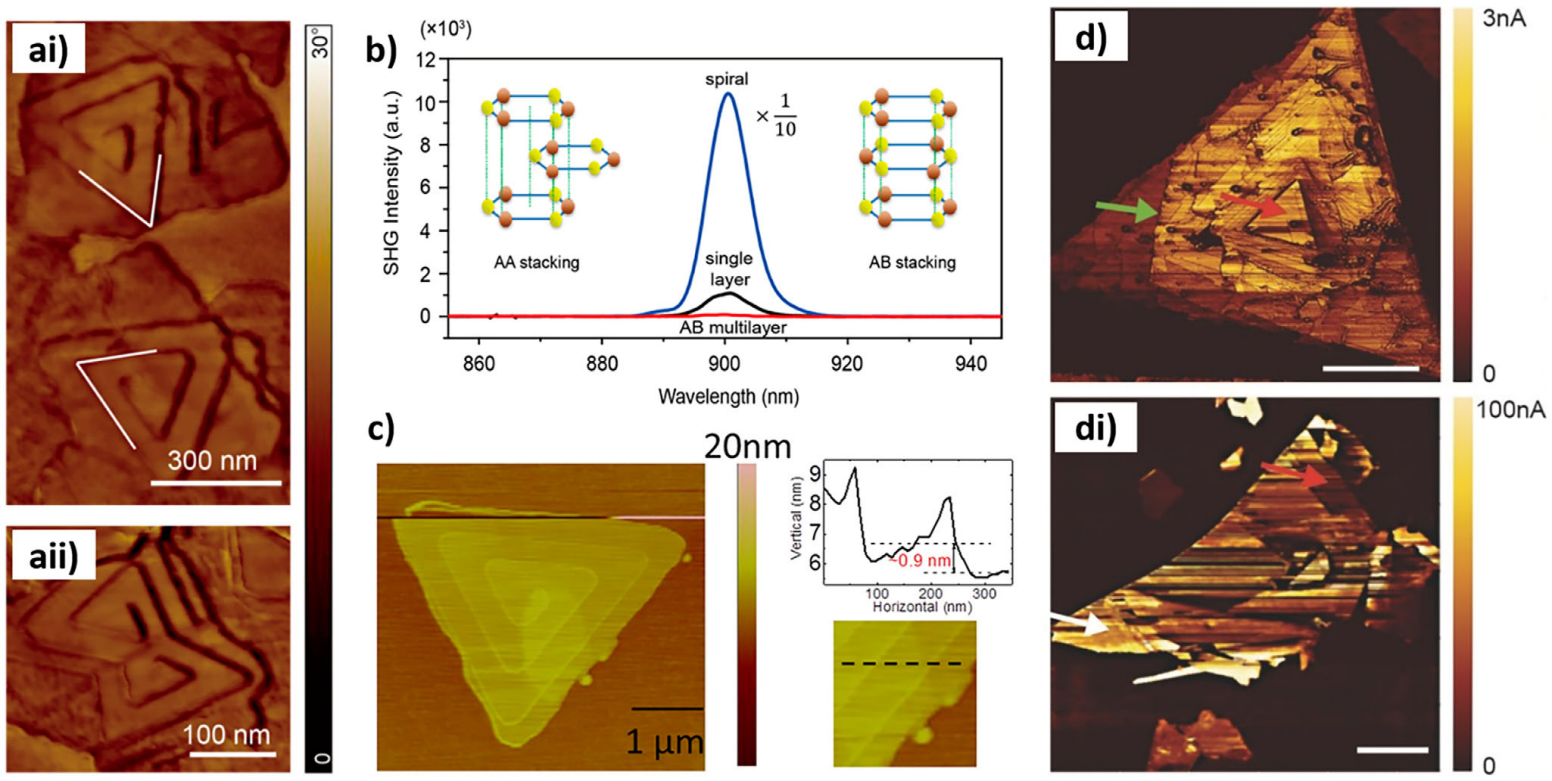
ai, aii) AFM images of MoS_2_ spirals showing different chirality. b) comparison of the SHG output of multilayer, single‐layer and spiral MoS_2_. (c) AFM characterization of a spiral WSe_2_ flake. Comparison of the electrical conductivity of MoS_2_ spiral (d) and multilayer MoS_2_ produced by exfoliation (di) using c‐AFM. (a, b) Adapted with permission from Zhang et al.,^[^ ^171^ ^]^ copyright 2014 American Chemical Society (c) adapted with permission from Chen et al.,^[^ ^179^ ^]^ copyright 2014 American Chemical Society and (d) adapted with permission from Ly et al.^[^ ^172^ ^]^

The formation of 3D spiral TMD flakes by SDD growth is well described by the Bruce‐Cabrera‐Frank (BCF) model of crystal formation.^[^ ^181^ ^]^ Screw dislocations are line defects characterized by a component of the displacement vector (Burgers vector) normal to the crystal face from which they emerge. Since screw dislocations behave as self‐perpetuating steps, the crystal growth does not require further nucleation.^[^ ^182^ ^]^ For this reason, SDD growth is particularly favorable in low supersaturation conditions relative to layer‐by‐layer (LBL) and dendritic growth.^[^ ^181^, ^182^, ^183^ ^]^ TMD nanocrystal growth via SDD is typically characterized by a 3D flat spiral morphology (Figure 10a), and the morphological chirality of these nanostructures is associated with the handedness of the spiral. However, in contrast with solution‐phase methods, vapor‐phase methods like CVD and related syntheses lack control over the symmetry breaking‐forming both clockwise and counter‐clockwise spirals at the same time (Figure 10b,c).^[^ ^171^, ^175^, ^182^ ^]^ Despite the lack of enantioselectivity, some degree of control on the flake morphology has been observed with a precise selection of the synthetic conditions. In particular, spiral TMD nanostructures were observed in a broad variety of morphologies, among which, the triangular spiral seems to be the most commonly observed. Hexagonal and truncated triangular spirals (Figure 11d) have also been documented along with complex supertwisted spirals (Figure 10e,f) and more complex morphologies.

The pioneering works from Zhang et al.^[^ ^171^ ^]^ and Chen et al.^[^ ^179^ ^]^ documented the first observations of TMD nanostructures produced by SDD growth, reporting on CVD methods to grow spiral MoS_2_ and WSe_2_ flakes. In particular, Zhang et al.^[^ ^171^ ^]^ observed the formation of 3D spiral MoS_2_ flakes via CVD synthesis using MoO_3_ and sulfur powder as precursors. The authors suggested that a spike in the nucleation rate during the initial step of the nanocrystals’ growth was crucial to promote the formation of screw dislocations. In this way, the formation of crystals with spiral morphology was achieved with a high yield (up to 80%) for a large range of substrates (SiO_2_/Si, mica, fused silica, and TiO_2_). AFM characterizations (Figure 11a) revealed a lateral size of around 300 nm measured from the edges of the largest basal plane, while each step caused an increase in the height profile of around 0.62 nm, in line with the thickness of a single MoS_2_ layer. HR‐TEM and computational investigation suggested an AA stacking in the spiral crystal, which breaks the inversion symmetry of bulk 2H‐MoS_2_. Interestingly, the authors observed an enhancement of the second harmonic generation (SHG) output of two orders of magnitude for the spiral MoS_2_ flake with respect to the monolayer (Figure 11b). Moreover, the SHG output was observed to increase with the layer number, further confirming the breaking of the inversion symmetry. Chen et al.^[^ ^179^ ^]^ observed the formation of spiral WSe_2_ nanocrystals using a sulfur‐assisted CVD synthesis. In this case, the addition of sulfur to the synthesis had the fundamental role of enhancing the reactivity of the selenium, promoting the selenization of WO_3_, analogous to the use of hydrogen as previously proposed by Huang and co‐workers.^[^ ^184^ ^]^ Morphological characterizations revealed the presence of triangular spiral flakes with basal dimensions from 3 to 5 µm (Figure 11c), and the thickness of the crystals was observed to be particularly sensitive to the growth temperature, favoring the growth of thicker crystals at higher temperatures, along with the formation of a small percentage of hexagonal flakes. In this case, the authors proposed that the low supersaturation achieved using the sulfur‐mediated synthesis favored SDD growth while suppressing the formation of nanocrystals via LBL growth.

Encouraged by these first observations, several other authors investigated the formation of spiral TMDs nanocrystals. Ly et al.^[^ ^172^ ^]^ reported detailed observations with TEM and dark field‐TEM (DF‐TEM) on the structure of spiral MoS_2_ nanocrystals, thanks to which the rhombohedral stacking structure was identified. Moreover, the investigation of the electrical properties of spiral MoS_2_ nanocrystals by c‐AFM measurements (Figure 11d), showed an almost constant electrical conductivity profile for the spiral flake at different heights, which is in stark contrast with the behavior observed for a stacked crystal produced by mechanical exfoliation that shows a rapid drop in conductivity with the sample height. The formation of spiral WS_2_ nanocrystals was reported by Sarma et al.^[^ ^175^ ^]^ using a CVD‐based sulfidation of WO_3_ particles drop‐casted on Si/SiO_2_ substrate. The analysis of the crystal's morphology observed by AFM revealed a broad distribution of flake morphologies comprising single‐layer and different stacking types. Interestingly, the comparison of photoluminescence properties between crystals grown by SDD and LBL mechanisms revealed the absence of the indirect recombination peak at around 900 nm for the spiral flake, which was related to possible metallic edges. Shearer et al.^[^ ^180^ ^]^ reported the observation of spiral WSe_2_ nanocrystals with multiple morphologies, such as triangular, hexagonal, and truncated triangular flakes (Figure 10d), along with more complex nanostructures produced by multiple screw dislocations. The authors related the different morphologies to the presence of multiple stacking layers, affecting the local symmetry and optical properties. In particular, SHG measurements suggested the breaking of inversion symmetry due to the stacking observed in triangular flakes, while hexagonal flakes restored the inversion symmetry and thus were observed to be SHG silent, and truncated triangular flakes showed a mixed behavior.

**Figure 12:**
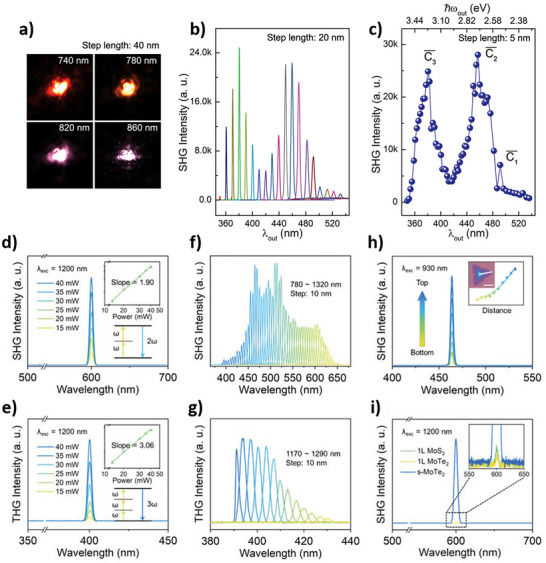
Investigation of the SHG properties of chiral TMDs nanospirals. a) CCD images of an R‐spiral of MoSe_2_ illuminated using different wavelengths (740, 780, 820, and 680 nm). b) SHG signal and SHG intensity (c) under a broad range of the excitation wavelengths (700 – 1080 nm) of R‐spiral of MoSe_2_. NLO response of spiral MoTe_2_, SHG (d), and THG (e) output under different excitation power and SHG (f) and THG (g) intensity output under different excitation wavelengths. SHG output of spiral MoTe_2_ observed at different heights (h) and comparison with monolayer MoS_2_ and MoTe_2_ specimens (i). (a‐c) Reproduced with permission of Wang et al.,^[^ ^173^ ^]^ and (d‐i) are reproduced with permission of Ouyang et al.^[^ ^174^ ^]^

**Figure 13:**
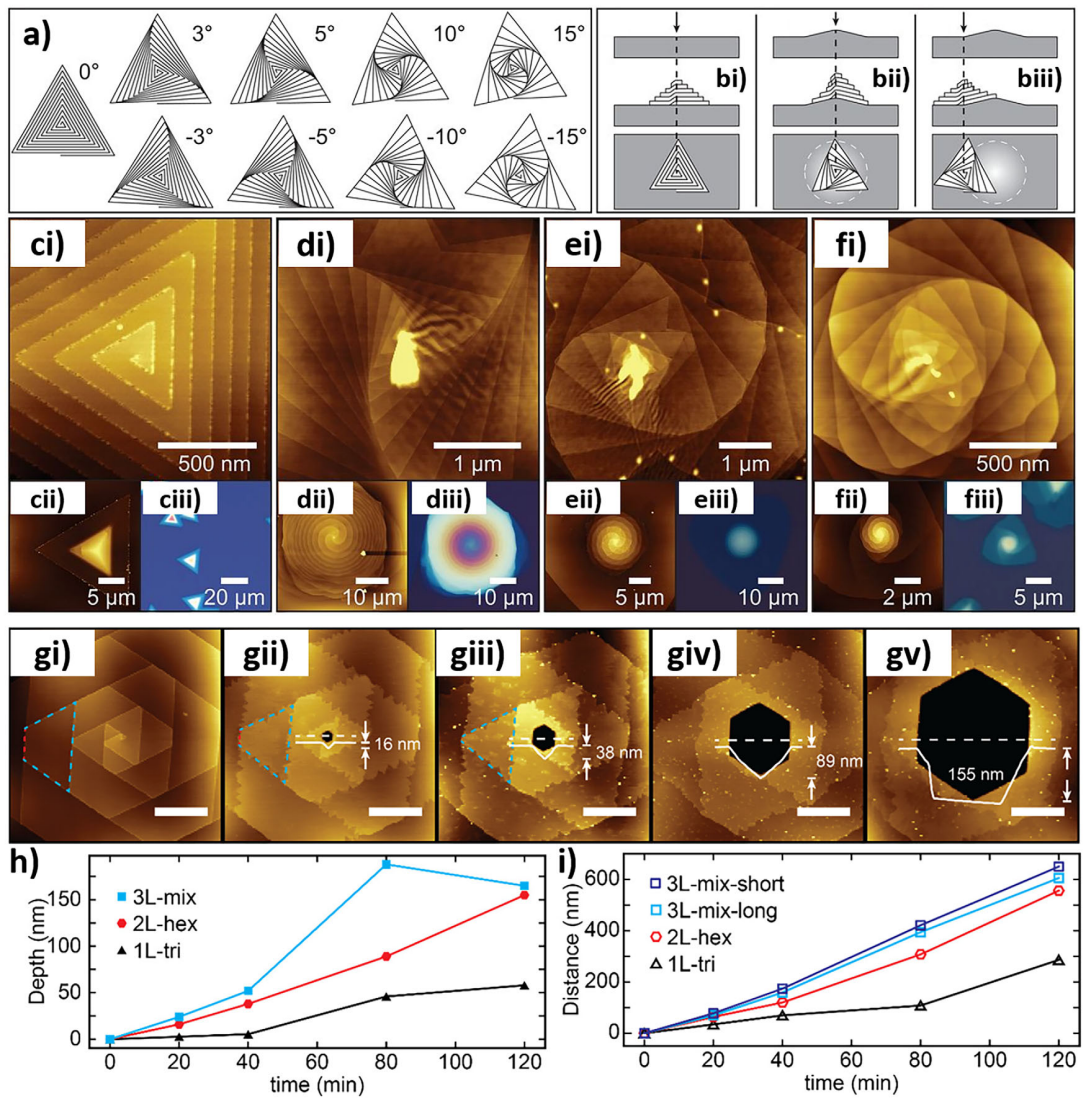
a) Simulated supertwisted spirals with increasing twist angle. b) Effect of the substrate surface on the formation of supertwisted spirals: flat spiral (bi), fastened supertwisted spiral (bii), and unfastened supertwisted spiral (biii). AFM (ci‐fi and cii‐fii) and optical microscopy (ciii‐fiii) images of different examples of WS_2_ supertwisted spirals with various twist angles. AFM characterization of the morphological evolution of a two‐layer WSe_2_ hexagonal spiral during etching with hydrogen peroxide: before etching (gi), 20 minutes (gii), 40 minutes (giii), 80 minutes (giv), and 120 minutes (gv), scale bars = 500 nm for all the images. Evolution of the hole depth (h) and distance between the center of the hole to its edge (i) observed at different etching times. (a‐f) adapted with permission from Zhao et al.^[^ ^176^ ^]^ and (g‐i) adapted with the permission from Zhao et al.^[^ ^177^ ^]^

The preparation of spiral MoSe_2_ and MoTe_2_ flakes was reported by Wang et al.^[^ ^173^ ^]^ and Ouyang et al.,^[^ ^174^ ^]^ respectively, along with an in‐depth investigation of the nonlinear optical properties of these twisted nanostructures. Using water vapor‐assisted atmospheric pressure chemical vapor transport (APCVT), Wang et al.^[^ ^173^ ^]^ investigated the growth of L and R spiral MoSe_2_ nanocrystals on SiO_2_/Si substrate starting from MoSe_2_ powder. Water vapor‐assisted CVT syntheses of TMDs were first introduced by Zhao et al.^[^ ^185^ ^]^ In these approaches, water vapor plays multiple critical roles during the transport process, including facilitating the volatilization and dissolution of the TMD precursors to form active metal oxyhydroxide and hydrogen chalcogenide species that take part in the crystal growth.^[^ ^185^ ^]^ Triangular spiral MoSe_2_ flakes showed remarkably high SHG due to their twisted 3R structure, which caused the breaking of inversion symmetry in the multi‐layer crystals (Figure 12a). Combining the increasing of the interaction volume in the multi‐layer systems and the intrinsic high second nonlinear susceptibility of MoSe_2_,^[^ ^186^ ^]^ spiral MoSe_2_ flakes showed extremely interesting performances as SHG emitters, with three orders of magnitude enhancement of the SHG intensity and broad excitation range (Figure 12b,c). Similarly, Ouyang et al.^[^ ^174^ ^]^ produced triangular spiral nanopyramids of MoTe_2_ with twisted 3R structure via CVD on SiO_2_/Si substrates pretreated with 4% HF solution to increase the surface roughness, a step deemed critical by the authors to promote the SDD growth. The spiral MoTe_2_ nanocrystals showed SHG (Figure 12d) and third harmonic generation (THG) activity (Figure 12e) over a broad range of frequencies (Figure 13f,g). Furthermore, the analysis of the SHG intensity revealed a strong dependence on the excitation frequency, with local maxima observed at 2.05, 2.41, and 2.67 eV (Figure 12f) which were associated with the B’, C, and D excitons, respectively.^[^ ^187^ ^]^ The investigation of the SHG output dependence with the layer number showed an increase with the number of layers (Figure 12 h) and the comparison of the SHG performance with MoS_2_ and MoTe_2_ monolayer (Figure 12i) revealed an enhancement of three orders of magnitude of the SHG intensities in the case of spiral MoTe_2_ nanocrystals.

Other morphologies like supertwisted spirals were achieved by growing spiral TMDs nanocrystals on non‐Euclidean surfaces, while etched spirals were produced using chemical etching in a post‐synthetic etching treatment to produce a complex holed nanostructure.^[^ ^176^, ^177^ ^]^ In investigations by Zhao et al.^[^ ^176^ ^]^ SiO_2_/Si substrates coated with SiO_2_ or WO_3_ particles (with diameters of hundreds of nanometers) were used to produce conical surfaces. WS_2_ and WSe_2_ flakes were grown via water vapor‐assisted CVT. In these conditions, the growth of spiral TMD nanocrystals was affected by the protrusions created by the conical surface. In particular, the characteristic spiral flakes produced by SDD growth were observed to undergo further twisting, leading to the formation of supertwisted nanostructures (Figure 13a). Both fastened and unfastened supertwisted spirals were produced by this approach. The authors observed that the type of superstructure produced was related to the relative position of the screw dislocation in relation to the conical surface (Figure 13b), and a large variety of morphologies characterized by different twisting angles could be distinguished (Figure 13c–f). Recently, Tong et al.^[^ ^178^ ^]^ investigated the SHG activity of WS_2_ supertwisted spirals with different twisting angles. In contrast with a triangular flat spiral, in which SHG increases with increasing the number of layers, as expected due to the lack of inversion symmetry in the 3R lattice, the supertwisted spirals showed a more complex behavior where the maximum intensity depended on the twisting angle. Finally, the chemical etching of spiral WS_2_ and WSe_2_ nanocrystals was investigated by Zhao et al.^[^ ^177^ ^]^ as a promising strategy to achieve further control of nanostructure morphology. In particular, treatment in hydrogen peroxide solution was used for the etching of triangular, hexagonal, and truncated triangular spiral, revealing the formation of holed structures in all cases examined (Figure 13g,h).

**Figure 14:**
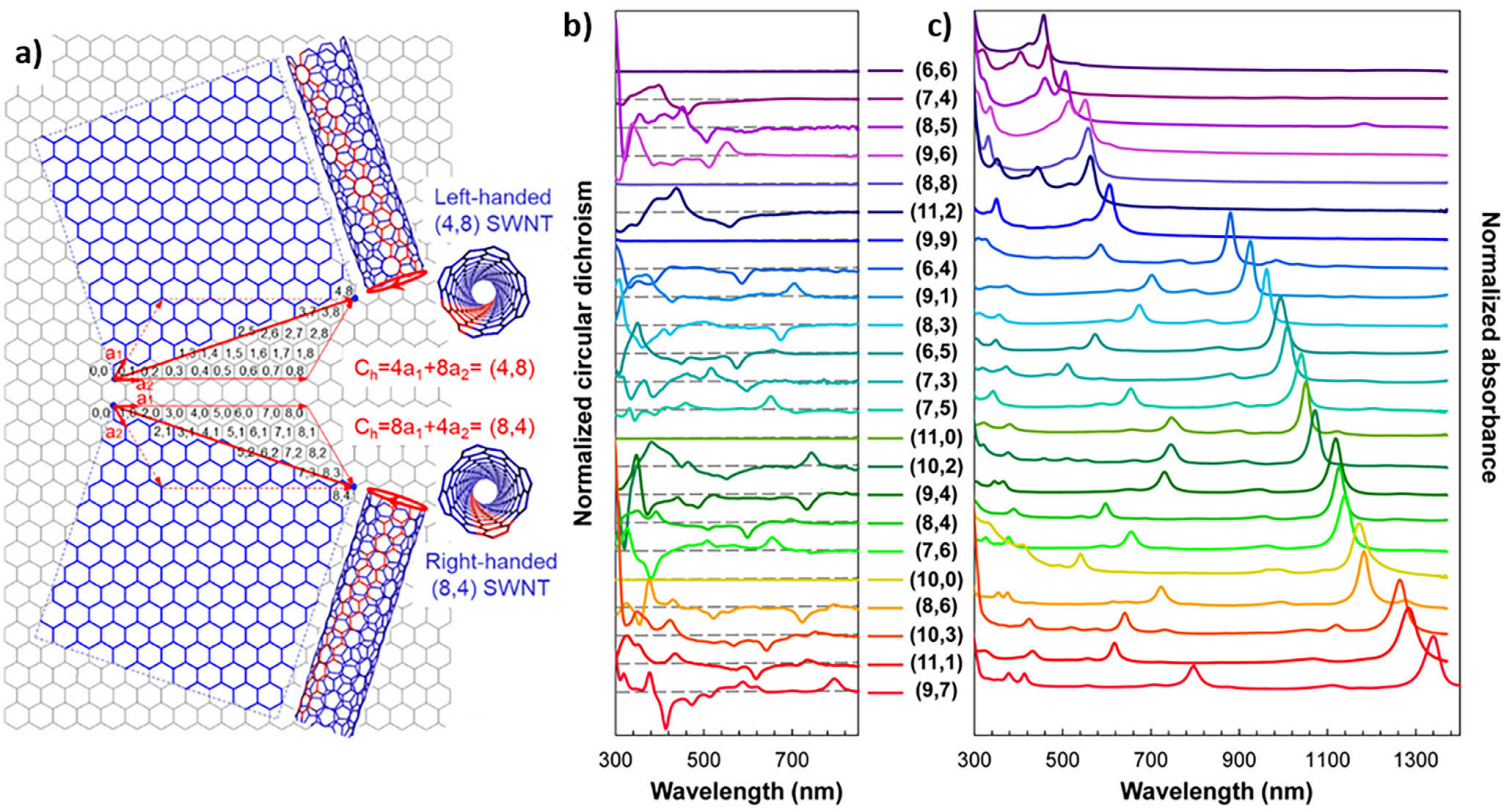
a) schematic of right‐handed and left‐handed carbon NTs assembled from graphene sheets rolled up along (8,4) and (4,8) vectors. b) CD spectra of single‐enantiomer single‐walled carbon NTs of varying chiral indices and c) corresponding UV‐Vis‐NIR spectra of single‐walled carbon NT dispersion. (a) adapted with permission from Yang et al.^[^ ^189^ ^]^, (b,c) adapted with permission from Ao et al.^[^ ^193^ ^]^

1D TMD nanotubes (NTs) are another fascinating example of nanostructure in which chirality can be observed. The chirality of an individual single‐walled nanotube (NT) is defined by a pair of chiral indices (*n, m*) specifying a chiral vector, where *n* and *m* represent multiples of the unit cell vectors for the underlying nanosheet (Figure 14a) from which the NT can be considered rolled up. Note that for the remainder of this section, “chirality” refers to these chiral vectors. Nanotubes are considered right‐handed and left‐handed if (*n – m*) > 0 and (*m – n*) > 0, respectively (Figure 14a). Research on chirality in TMD NTs is still in its early stages, and just a few works deal with the identification of the structural isomers, and the effect of these geometric characteristics has not yet been reported. This is hampered by the challenge of producing TMD NTs with controllable chirality along with their typical multi‐layer structure.^[^ ^103^ ^]^ Only recently, An et al.^[^ ^188^ ^]^ reported a synthesis for the production of WS_2_ NTs with single chiral angles by carefully controlling the temperature during the CVD growth. Despite the many differences, we can consider an analogy with carbon NTs, where a deeper level of understanding of the effect of chirality has been achieved thanks to the large amount of work dedicated during the last decades to synthesis, purification, and characterization.^[^ ^189^, ^190^, ^191^, ^192^ ^]^ In this case, NT chirality has been shown to greatly affect electronic and optical properties of NTs (Figure 14b,c). Furthermore, even highly enantiomer‐enriched carbon NT dispersions exhibit markedly different chiroptical activities depending on their chirality (Figure 14b), thus fine control of both chirality and enantiomeric excess is required for any application of chiral NTs.

**Figure 15:**
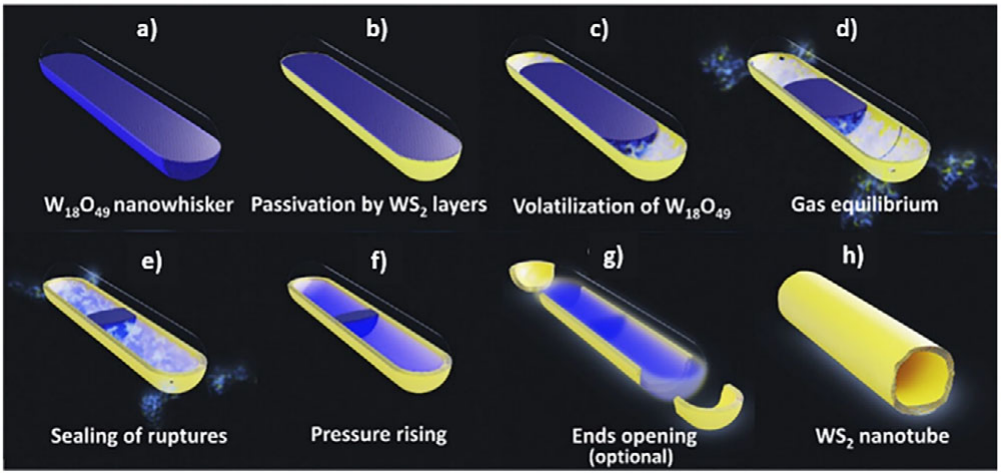
Growth mechanism of WS_2_ NTs on W_18_O_49_ nanowhiskers. NT is shown as midsection model in panels a–h. Reproduced with permission from Kundrát et al.^[^ ^196^ ^]^

Since the first observation of the formation of multi‐walled WS_2_ NTs from Tenne et al.^[^ ^102^ ^]^ in 1992, several efforts were dedicated to the optimization of the TMD NT synthesis and understanding of the processes involved in the NT growth.^[^ ^194^, ^195^ ^]^ In a recent study, Kundrát and coworkers^[^ ^196^ ^]^ used in situ SEM monitoring of vapor‐phase growth of multi‐walled WS_2_ NTs on W_18_O_49_ nanowhiskers using H_2_S/H_2_ mixture. Two main growth mechanisms were observed: the well‐established “surface‐inwards” mechanism and a novel “receding oxide core” mechanism (Figure 15). Initially, several layers of WS_2_ are formed rapidly on the outside of the tungsten suboxide layer (Figure 15a,b), forming the exterior of the final NT via the surface‐inwards mechanism. Subsequently, receding oxide core growth predominates (Figure 15c–h). The oxide core reacts with H_2_ and is preferentially volatilized along the <010> direction (Figure 15c,d), gradually etching the oxide core. Metal oxide vapor then reacts with H_2_S within the cavity to form WS_2_ vapor, which is then deposited as new layers on the inner NT surface and sealing defects in the NT surface. Finally, at sufficiently high temperatures, pressure buildup can open the NT ends (Figure 15g) to yield a typical multi‐walled WS_2_ NT (Figure 15h).

As briefly mentioned before, the recent report from An et al.^[^ ^188^ ^]^ presented a direct and facile growth of WS_2_ NTs with a predominant contribution of a single chiral angle. This was achieved by implementing a different synthesis approach using gold nanoparticles as catalysts for the CVD reaction. The authors observed that the gold NPs provide a unique site for the WS_2_ nucleation and growth, giving very different control over the NTs structure compared to the most common WO_3‐x_ sulfurization procedure.^[^ ^197^, ^198^, ^199^, ^200^ ^]^ In particular, the authors observed that the NT's growth process is highly sensitive to the temperature of the substrate. Selected area electron diffraction (SAED) revealed a single set of hexagonally arranged diffraction spots for multi‐walled WS_2_ NTs prepared at 835–840 °C, confirming the coherent helical angle for all the concentric layers. The analysis of the chiral angle distribution revealed almost 79% of the sample was composed of single chiral angle NTs with a preference for achiral zigzag and armchair configurations. By contrast, the presence of multiple chiral angles was observed for NTs produced at 840–845 °C.

**Figure 16:**
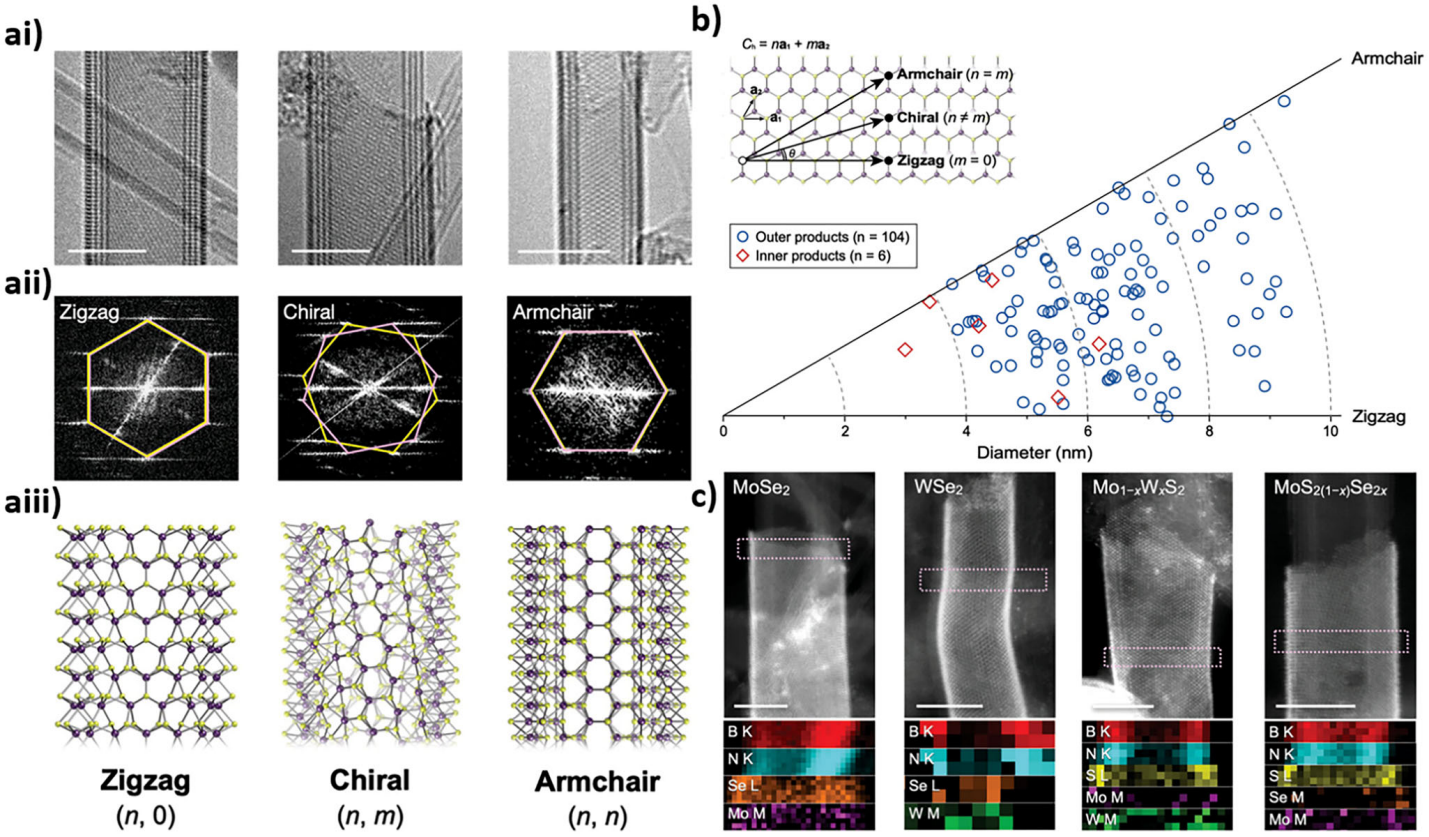
a) HR‐TEM of single‐walled MoS_2_ NTs growth on BNNTs showing different chiral angles, scale bar 5 nm. aii) The corresponding FFT analysis revealing the chiral angles of 0.0°, 15.7°, and 30°. aiii) structural model of MoS_2_ NTs with zigzag, chiral and armchair structures. b) Distribution of the chirality for MoS_2_ single‐walled NTs growth on BNNTs. c) characterization of other TMD NTs produced by template growth on BNNTs: High‐angle annular dark‐field scanning TEM (HAAD STEM) scale bars = 5 nm, electron energy loss spectroscopy (EELS), and energy dispersive spectroscopy (EDS) mapping. Adapted with permission from Nakanishi et al.^[^ ^202^ ^]^

A promising strategy for the production of single‐walled MoS_2_ NTs was reported by Xiang et al.^[^ ^201^ ^]^ relying on the use of 1D nanostructures such as boron nitride NTs (BNNTs) as a template for TMD growth. Moreover, this strategy was adopted to grow even more complex ternary 1D van der Waals heterostructures based on single‐walled carbon NTs‐BN‐MoS_2_. A similar approach for the production of TMD NTs with superior structural control was reported by Nakanishi et al.^[^ ^202^ ^]^ where, even in this case, BN NTs were used as substrate to grow high‐quality single‐walled MoS_2_ NTs with a diameter from 4 to 10 nm (Figure 16ai). FFT analysis of the HR‐TEM was used to obtain the chiral angle of MoS_2_ NTs (Figure 16aii,aiii). The population of the NTs’ chiral angles (Figure 16b) was compared with a statistical model, revealing the production of NTs with mostly random chiral angles, thus, no obvious selectivity on the chirality of the TMD NTs formed on the BN NT template. Furthermore, the authors used this approach to grow a variety of single‐wall TMD NTs, pure phases (MoS_2_, MoSe_2_, WSe_2_), alloys Mo_1‐x_W_x_S_2_ and Janus NTs (MoS_2(1‐x)_Se_2x_) (Figure 16c).

Vapor‐phase syntheses based on CVD, CVT, and related approaches have proven unique for the production of TMD nanostructures with complex chirality and superior control of morphology and crystallinity. The investigation of spiral TMD flakes formed via SDD growth revealed critical details on the formation of these fascinating chiral nanostructures.^[^ ^171^, ^179^ ^]^ Moreover, properties related to their characteristic structure have been observed, such as the enhanced NLO response, as observed both in SHG and THG, and vertical electrical conductivity.^[^ ^172^, ^173^, ^174^ ^]^ More complex structures, such as supertwisted spirals obtained by controlling the SDD growth on a non‐Euclidean surface and etched spiral TMD flakes, further expand the range of complexity reachable with these approaches.^[^ ^176^, ^178^ ^]^ However, these approaches still lack enantioselectivity, and racemates containing different enantiomers are produced with equal probability.^[^ ^171^, ^175^, ^179^ ^]^ Achieving such control on the crystal morphology would allow for the investigation of the chiroptical properties as well as the production of highly chiral films with promising applications in several fields, particularly in photonics and sensing.

1D TMD NTs grown by CVD can show structural chirality associated with their chiral angle. In contrast to carbon NTs, TMDs form NTs with larger diameters and typically multi‐walled structures. This was related to the strain energy associated with rolling up the three‐atom‐thick monolayer of TMDs with respect to the single‐atom carbon layer.^[^ ^201^, ^203^ ^]^ In addition to the challenges of synthesizing single‐chirality NTs, the difficulties of enantioselective synthesis of TMD NTs present a significant barrier to their implementation. Due to the high temperatures used (typically > 800 °C), symmetry breaking with organic ligands is clearly unfeasible for TMD NT synthesis. The higher thermal stability of inorganic templates may be a more feasible alternative. In particular, SiO_2_ nanowires have also been used as templates for the growth of MoS_2_ NTs,^[^ ^204^ ^]^ and a number of approaches for the synthesis of highly chiral silica nanowires and nanoribbons have emerged in recent years. On the other hand, post‐synthetic separation represents a more likely method for resolving complex mixtures of TMD NTs with different chirality.^[^ ^202^ ^]^ Stereoselective synthesis and separation of carbon NTs have been the subject of several reviews,^[^ ^189^, ^191^, ^205^ ^]^ however, these approaches remain comparatively unexplored in TMD NTs. Methods for the isolation of optically active carbon NTs using optically pure ligands have been known since 2007.^[^ ^206^ ^]^ In recent years, separation using gel column chirality and aqueous two‐polymer phase extraction has allowed for the sorting of single enantiomers.^[^ ^207^, ^208^, ^209^ ^]^ While no such approaches have yet been reported for TMD NTs, their increased surface polarity may render such approaches more feasible than in carbon NTs.

## 4 Summary and Outlook

Following this review on the emergence of chirality in TMD nanostructures, it is evident that chirality plays a significant role in the development of novel TMD nanostructures with unique properties and a broad range of potential applications. Solution‐phase TMD nanostructures have shown promising results in their application in nanomedicine both invitro and invivo. Promising activities such as angiogenic activity and reprogramming of the macrophage population in tumor microenvironment, along with ROS detection have been reported.^[^ ^121^, ^125^, ^126^ ^]^ Enantioselective catalytic activity has been observed for chiral TMDs QDs. The CISS effect was observed in chiral hybrid TMD system, a promising feature for spintronics and spin‐dependent electrocatalysis.^[^ ^114^, ^116^, ^119^ ^]^ TMD nanostructures synthesized by vapor‐phase methods like spiral TMD flakes produced by SDD growth and the related supertwisted spiral have shown exciting NLO performances with enhancement of SHG of orders of magnitude with respect to monolayer systems which makes these systems suitable for application in NLO devices.^[^ ^171^, ^174^, ^178^ ^]^ Various synthetic techniques have been successfully employed to prepare a wide range of nanostructures, from 0D QDs, 1D NTs and 2D nanoflakes, to 3D spirals, and hybrid superlattices, along with colloidal nanoparticles with complex morphologies. Distinct types of chiral origins can be observed; colloidal systems produced via solution‐phase methods rely mostly on ligand‐induced chirality, where the functionalization with a chiral ligand affects the electronic transition of the achiral particles, and some examples of chiral morphology are reported as well. TMD nanostructures produced in the vapor‐phase show characteristic chiral morphology in the case of spiral TMD flakes formed by SDD growth or chiral structure in the case of TMD NTs.

Solution‐phase approaches have been used to produce chiral 0D QDs and more complex 3D nanostructures via bottom‐up methods, and chiral 2D nanostructures via top‐down methods. These methods offer appealing control of symmetry breaking through the introduction of chiral ligands, and possible biomedical applications of the resulting dispersions have been subject to significant attention. However, caution should be taken when investigating the contribution of impurities; contamination by chiral coordination complexes, organic molecules, and carbon dots should be properly investigated in relation to the specific synthetic approach and crystal growth mechanism. Moreover, care must be taken in the assignment of the chiroptical activity in relation to the specific electronic transitions that are involved, as well as the investigation of the origin of chirality in different systems. Potential artifacts such as linear dichroism and linear birefringence can affect CD measurements and must be properly evaluated, especially for the investigation of anisotropic systems and films. The analysis of the linear dichroism or relying on Mueller matrix polarimetry could reveal the presence of multiple contributions in the CD spectra. Recent advances in highly chiral hybrid MoS_2_ and hydrothermally produced MoS_2_ are expected to have a significant impact on future studies of solution‐phase TMD nanostructures. Bottom‐up approaches are particularly suitable for scale‐up and future investigation using continuous flow synthesis would further project these materials toward their industrial application.^[^ ^210^ ^]^ Since the original report from Liu et al.^[^ ^95^ ^]^, the hydrothermal synthesis of 1T‐MoS_2_ was originally designed for gram‐scale production, and the design of hydrothermal continuous flow syntheses of achiral TMDs nanostructures has already been proven.^[^ ^211^, ^212^, ^213^ ^]^ Vapor‐phase approaches, particularly CVD, have proven highly useful for the synthesis of 1D nanotubes and complex 3D spiral structures with high crystallinity and controllable morphology. These nanostructures have highly desirable properties for electronics and optoelectronics applications; however, their chirality‐dependent behavior remains a barrier to their implementation. Enantiomeric control of growth and separation of nanotube chirality may introduce further applications for these nanostructures. The use of hard chiral inorganic templates for selective growth may prove fruitful.

A lack of theoretical models continues to hinder a deeper understanding of chiroptical activity and its relationship with the chirality of the TMD nanostructures. Most of the work done so far relies on models designed for semiconductor QDs, and dubious or misleading interpretation of the chiroptical activity is often observed. Further efforts on this aspect will substantially contribute to the future development of chiral TMD nanostructures and their applications. The large variety of chiral TMD nanostructures, from QDs to micron‐scale assemblies, requires a diverse range of computational methodologies for different scenarios. Atomistic modelling of TMD QD‐ligand interfaces via density functional theory and/or molecular dynamics simulations should help to resolve ambiguity surrounding the origin of chiroptical activity in these systems, similar to previous investigations of binary QD systems.^[^ ^35^, ^214^ ^]^ For larger nanostructures such as nanosheet assemblies, classical electrodynamics methods (e.g., finite difference time domain, coupled dipole approximation, finite element integration, etc.) have been used to successfully model CD spectra for a wide range of nanomaterials.^[^ ^38^, ^43^, ^215^ ^]^ We expect such computational modeling to afford greater understanding of the chiroptical properties of TMD nanostructures and aid in their design moving forward.

Moreover, most of the research to date has been focused on group VI TMDs. We believe that future works in the field will concentrate on the design of novel synthetic techniques capable of extending the palette of chiral TMD nanostructures toward other less‐investigated combinations, alloys, and heterostructures. Moreover, particular attention should be devoted to assessing the effect of chirality and chiroptical activity according to the different nanostructure morphology and polymorphs, extending the chirality of TMDs nanostructure to a wide range of materials with different electronic properties.

## Conflict of Interests

The authors declare no conflict of interest.

## Data Availability

The data that support the findings of this study are available from the corresponding author upon reasonable request.
